# Downregulated annexin A1 expression correlates with poor prognosis, metastasis, and immunosuppressive microenvironment in Ewing’s sarcoma

**DOI:** 10.18632/aging.204615

**Published:** 2023-03-28

**Authors:** Xiangwen Shi, Yipeng Wu, Linmeng Tang, Haonan Ni, Yongqing Xu

**Affiliations:** 1 Kunming Medical University, Kunming, China; 2 Laboratory of Yunnan Traumatology and Orthopedics Clinical Medical Center, Yunnan Orthopedics and Sports Rehabilitation Clinical Medical Research Center, Department of Orthopedic Surgery, 920th Hospital of Joint Logistics Support Force of PLA, Kunming, China; 3 Bone and Joint Imaging Center, Department of Medical Imaging and Radiology, The First Affiliated Hospital of Hebei North University, Zhangjiakou, China

**Keywords:** bone tumor, inflammation, immunotherapy

## Abstract

Objective: Ewing’s sarcoma (ES) is a common bone malignancy in children and adolescents that severely affects the prognosis of patients. The aim of this study was to identify novel biomarkers and potential therapeutic targets for ES.

Methods: Highly prognosis-related hub genes were identified by independent prognostic analysis in the GSE17679 dataset. We then performed survival analysis, Cox regression analysis and clinical correlation analysis on the key gene and validated them with the GSE63157, GSE45544 and GSE73166 datasets. Differentially expressed genes (DEGs) were screened based on the high and low expression of key gene, Gene Ontology (GO), Kyoto Encyclopedia of Genes and Genomes (KEGG) pathway analyses, Gene Set Enrichment Analysis (GSEA), and Gene Set Variation Analysis (GSVA) were performed to explore the underlying mechanisms of ES, and significant module genes were established based on protein-protein interaction (PPI) networks. Furthermore, the correlations between module genes and the immune microenvironment were analyzed and the correlations between the key gene and immune infiltration levels in sarcoma were investigated using TIMER and TISIDB. Finally, the expression levels of these key genes in ES cell lines (RD-ES and A673 cells) were further validated by real-time quantitative PCR (RT-qPCR). CCK-8 and EdU assays were performed to assess the effect of ANXA1 knockdown on RD-ES cell proliferation.

Results: ANXA1 was identified as a key gene for ES prognosis. The overall survival (OS) time of patients with low ANXA1 expression was shorter, and the expression level of ANXA1 in the metastatic group was significantly lower than that in the primary group (P<0.01). Additionally, the abundance of 12 immune cells in the ANXA1 low-expression group was significantly lower than that in the high-expression group (all P<0.05), which may be related to the inhibition of the immune microenvironment. A PPI network was constructed based on 96 DEGs to further identify the five ANXA1-related module genes (COL1A2, MMP9, VIM, S100A11 and S100A4). The expression levels of ANXA1, COL1A2, MMP9, VIM, S100A11 and S100A4 were significantly different between ES cell lines and mesenchymal stem cells after validation in two ES cell lines (all P<0.01). Among these genes, ANXA1, COL1A2, MMP9, VIM and S100A4 were significantly associated with the prognosis of ES patients (all P<0.05). Importantly, ANXA1 knockdown significantly promoted the proliferation of RD-ES cells, which may explain the susceptibility to ES metastasis in the ANXA1 low-expression group.

Conclusions: ANXA1 may serve as an independent prognostic biomarker for ES patients and is associated with metastasis and the immunosuppressive microenvironment in ES, which needs to be validated in further studies.

## INTRODUCTION

Ewing’s sarcoma (ES) is the second most frequent and highly invasive bone tumor, accounting for 15% of all bone tumors in children and adolescents [1]. According to early data from the American Cancer Center, there are approximately three cases of ES per million people each year [2]. The incidence of ES is higher in white populations, and it is more common in males for unknown reasons [3, 4]. Despite being less than 1% of all human malignant tumors [5], sarcomas are all invasive, and the most common metastatic sites are the lung, bone, and bone marrow [6].

The standard treatment for ES includes a combination of surgery, local radiotherapy, and drug chemotherapy [7, 8]. After the introduction of chemotherapy, the survival rate of local ES patients increased from 10% to approximately 75%, but the metastasis of ES is still not optimistic [9, 10]. It has been reported that approximately 20-25% of patients with ES have metastases at the time of diagnosis, with a 5-year survival rate of less than 30% [11, 12]. In view of the above factors, new treatments are urgently needed to improve the prognosis of ES patients. In contrast to other sarcoma types, specific chromosome translocations such as EWS-TL11, are necessary conditions for Ewing’s sarcoma [13, 14]. Therefore, the pathological process controlled by the fusion protein determines the importance of molecular targeted therapy for Ewing’s sarcoma. Several previous studies evaluated indicators affecting ES survival and metastasis based on the SEER database and additional multicenter cohort data, and further developed nomogram models to predict the incidence of ES [15, 16]. Recently, the development of public databases and genomics has made it critical to seek potential prognostic biomarkers for ES, which would improve the prognosis of patients and guide their treatment.

In this study, we analyzed four datasets, GSE17679, GSE63157, GSE45544 and GSE73166, from the Gene Expression Omnibus (GEO). High prognosis-related genes in the GSE17679 dataset were screened by independent prognostic analysis, and survival analysis, Cox regression analysis and clinical correlation analysis were performed based on these genes. 88 ES samples from the GSE17679 dataset were used as the experimental group, and 107 ES samples from the GSE63157, GSE45544 and GSE73166 datasets were used as the validation group to validate the results of the survival analysis, independent prognostic analysis and clinical correlation analysis. Differential analysis, Gene Ontology (GO) and Kyoto Encyclopedia of Genes and Genomes (KEGG) enrichment analysis were performed with median expression values of hub gene. Additionally, a protein-protein interaction (PPI) network was constructed to identify the module genes associated with hub gene to further explore the relationship between these module genes and the ES immune microenvironment. Finally, the expression of module genes was validated in ES cell lines (RD-ES and A673 cells) and mesenchymal stem cells (MSCs), and survival analysis was conducted. Exploring the effect of ANXA1 knockdown on the proliferation of RD-ES cells by CCK8 and EdU assays.

## RESULTS

### Screening of hub genes

First, a total of 64 genes were screened by Cox regression analysis of patients with ES based on OS time and survival status (*P*<0.001) (Supplementary Table 1). Next, 50 genes were further obtained from 88 samples screened by survival analysis according to OS time and survival status (*P*<0.001) (Supplementary Table 2). Finally, 374 genes were significantly associated with at least one clinical characteristic through clinical correlation analysis, of which 50 genes were significantly associated with two clinical features, and 324 genes were significantly associated with only one clinical feature (Supplementary Table 3). Based on survival (*P*<0.001), independent prognostic (*P*<0.001), and clinical correlation analyses (SigNum=2), ANXA1 was determined to be the final hub gene.

### Survival analysis of ANXA1 and validation

**Figure 1:**
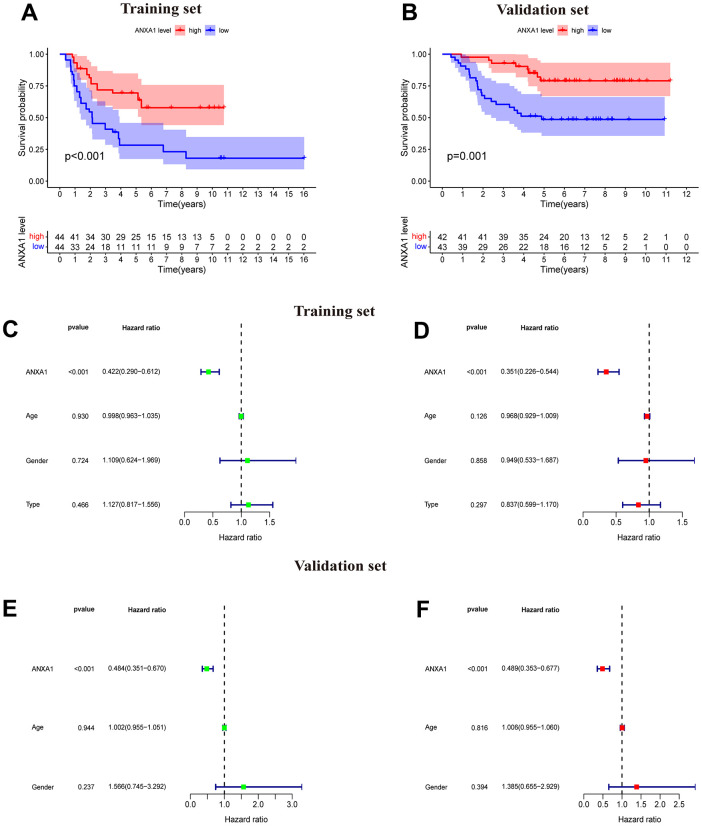
**ANXA1-based survival curves, univariate and multivariate Cox regression analysis.** (**A**) The results of Kaplan–Meier survival analysis based on the GSE17679 dataset suggested that patients in the ANXA1 low expression group had worse OS times than those in the low expression group (*P*<0.001). (**B**) The results of Kaplan–Meier survival analysis based on the GSE63157 dataset suggested that patients in the ANXA1 low expression group also had worse OS time, which was consistent with the training set (*P*=0.001). (**C**) Univariate Cox regression analysis for ANXA1 in the GSE17679 dataset (*P*<0.001). (**D**) Multivariate Cox regression analysis for ANXA1 in the GSE17679 dataset (*P*<0.001). (**E**) Univariate Cox regression analysis for ANXA1 in the GSE63157 dataset (*P*<0.001). (**F**) Multivariate Cox regression analysis for ANXA1 in the GSE63157 dataset (*P*<0.001).

A total of 88 samples in the training set were divided into a high-expression group and a low-expression group according to the median expression of ANXA1, and a survival curve was drawn. The results suggested that the OS time of patients with low-expression was significantly lower than that of patients with high-expression (*P* < 0.001), and the five-year survival rates of the ANXA1 low and high-expression groups were approximately 30% and 70%, respectively (Figure 1A). To validate the results of ANXA1 survival analysis, 85 samples from the GSE63157 dataset were divided into high and low-expression groups. According to the results, patients in the low-expression group had a shorter OS time than those in the high expression group (*P*=0.001), and the five-year survival rates of the ANXA1 low and high-expression groups were approximately 50% and 75%, respectively, which supported the conclusion of the experimental group (Figure 1B). To further analyze the prognostic value of ANXA1 in patients with different types of ES, survival analyses were performed in primary ES, recurrent ES, and metastatic ES patients from the GSE17679 dataset. The results showed that the OS time of the ANXA1 low expression group was significantly lower than that of the ANXA1 high expression group in primary (*P*<0.001) and metastatic ES patients (*P*=0.028), while there was no significant difference in recurrent ES patients (Supplementary Figure 1).

### Univariate and multivariate Cox regression analysis of ANXA1

The results of univariate analysis based on sex, age and tumor status suggested that ANXA1 was significantly associated with the survival and status of ES patients (*P*<0.001) and that the low HR value (HR<1) of ANXA1 indicated that it may be a low risk factor for prognosis in ES patients (Figure 1C). Multifactorial regression analysis indicated that ANXA1 was a low-risk factor for prognosis in ES patients (*P*<0.001) (Figure 1D). Univariate and multifactorial regression analyses from the validation group similarly demonstrated that ANXA1 could be an independent prognostic factor for predicting prognosis in ES patients (*P*<0.001) (Figure 1E, 1F).

### The expression of ANXA1 in clinical subgroups

**Figure 2:**
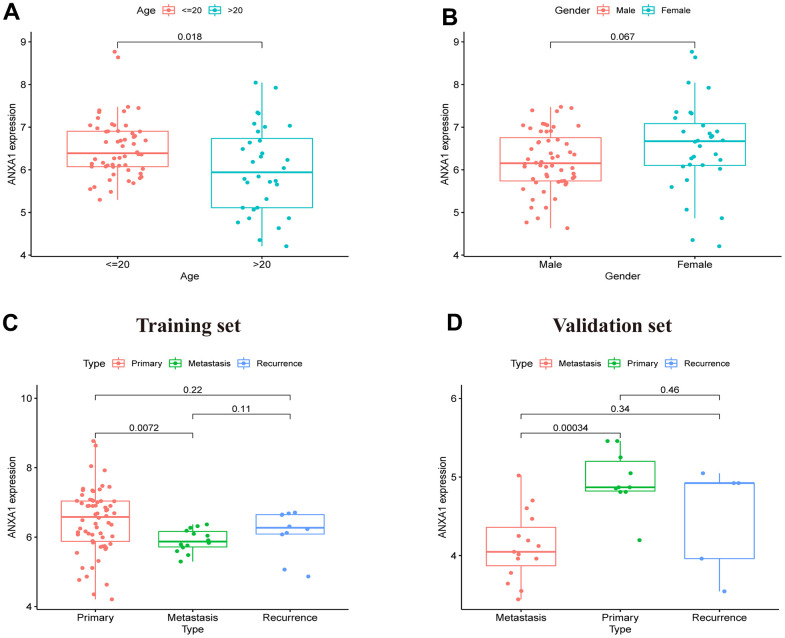
**ANXA1 expression in different subgroups based on the GSE17679, GSE45544 and GSE73166 datasets.** (**A**) In different age groups, the expression levels of the ANXA1 gene were higher in the ≤ 20-year-old group than in the > 20-year-old group (*P* = 0.018). (**B**) In different gender groups, the expression levels of ANXA1 gene were not significantly different (*P* = 0.067). (**C**) In different type groups, the expression levels of the ANXA1 gene were higher in the primary group than in the metastasis group (*P* = 0.0072), and there was no significant difference between the remaining groups. (**D**) 22 ES samples from the GSE45544 and GSE73166 datasets validated that the expression levels of the ANXA1 gene were higher in the primary group than in the metastasis group.

After dividing the clinical characteristics into specific subgroups, we analyzed the expression of ANXA1 in a clinical subgroup from the GSE17679 dataset. In the subgroup of age, the median expression of ANXA1 in the ≤20-year-old group was higher than that in the >20-year-old group (*P*=0.018) (Figure 2A). In the gender subgroup, there was no significant difference in ANXA1 expression levels between male and female patients (*P*>0.05) (Figure 2B). In the subgroup of tumor status, the median expression of ANXA1 in the metastasis ES group was significantly lower than that in the primary ES group (*P*=0.0072), but there was no significant difference between the primary group and recurrence group (*P*>0.05) (Figure 2C). Additionally, 22 ES patients from combined the GSE45544 and GSE73166 datasets were used to verify the age, gender and tumor status of the subgroup, and the results showed that the median expression of ANXA1 in the metastasis ES group was significantly lower than that in the primary ES group (*P*=0.00034) (Figure 2D).

### Identification and enrichment analysis of DEGs

**Figure 3:**
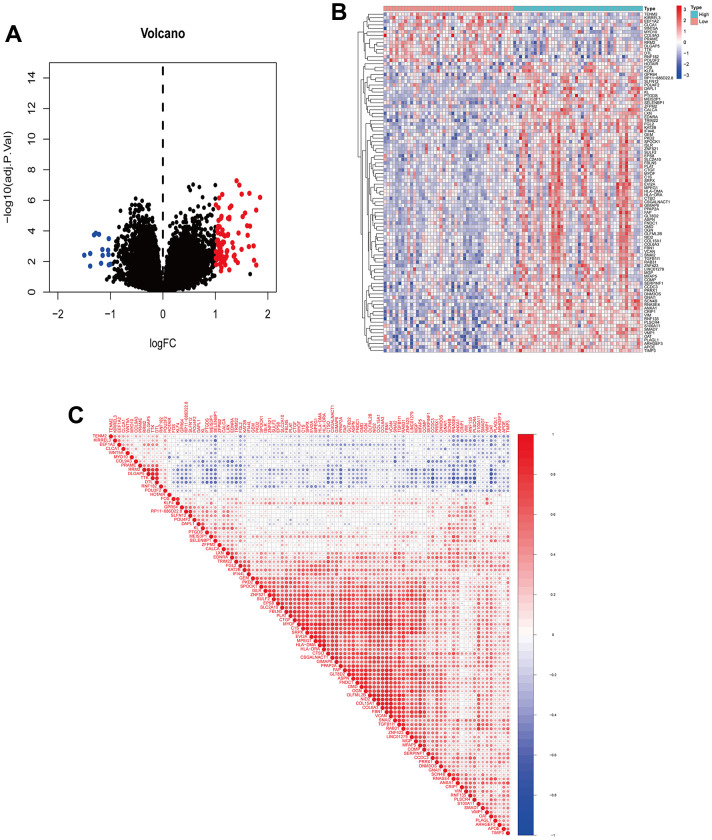
**Visualization of DEGs and correlation analysis of ANXA1.** (**A**) Volcano plot of DEGs. Red dots indicate upregulated genes, green dots indicate downregulated genes, and black dots indicate genes with insignificant differences. (**B**) Heatmap of DEGs. Red indicates high expression, blue indicates low expression, and white indicates moderate expression. (**C**) Correlation coefficient heatmap of DEGs. Red represents positive correlation and green represents negative correlation.

**Figure 4:**
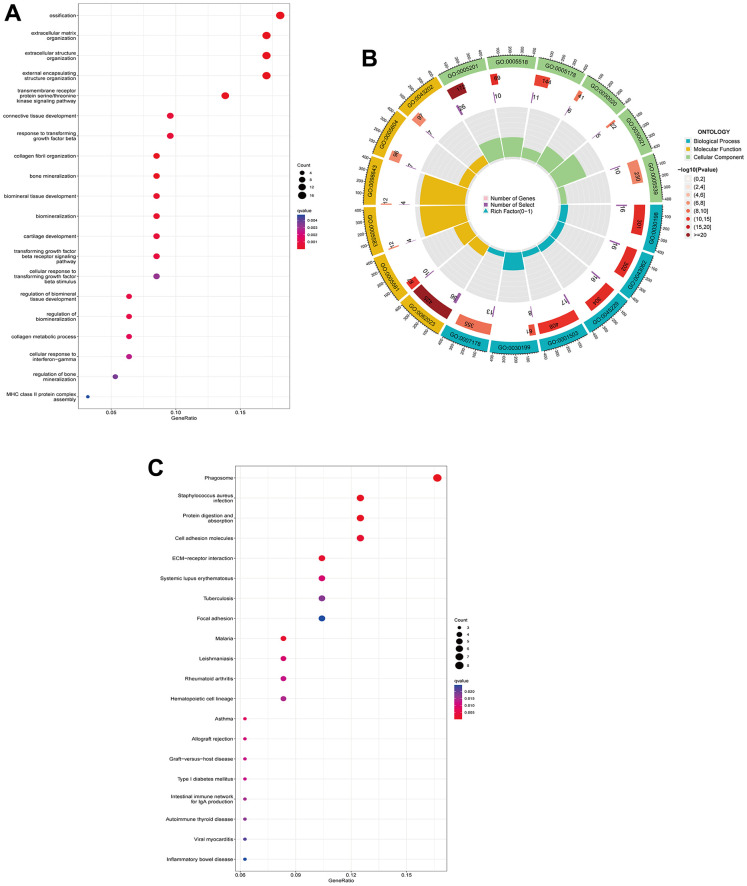
**GO functional annotation and KEGG pathway analysis of DEGs.** (**A**) The top 5 biological processes (BP), cellular components (CC), and molecular functions (MF) of DEGs. (**B**) Enrichment circle diagram of DEGs. (**C**) KEGG pathway analysis of DEGs.

A total of 95 DEGs were identified, of which 81 were upregulated genes and 14 were downregulated genes. The visual volcano plot and heatmap showed that the high-expression genes and the low-expression genes could be clearly distinguished according to the median expression of ANXA1 (Figure 3A, 3B). Further analysis of the correlation between ANXA1 and DEGs showed that 81 genes were positively correlated with ANXA1, and 14 genes were negatively correlated with ANXA1 (Figure 3C). In GO enrichment analysis (Figure 4A, 4B), DEGs were mainly involved in biological processes (BPs) such as the formation of extracellular matrix organization (16 genes), formation of extracellular structure organization (16 genes), ossification (17 genes), formation of external encapsulating structure organization (16 genes), and formation of collagen fibril organization (8 genes). In cellular components (CCs), DEGs were mainly involved in collagen-containing extracellular matrix (36 genes), collagen trimer (10 genes), fibrillar collagen trimer (4 genes), banded collagen fibril (4 genes), and basement membrane (7 genes). In terms of molecular functions (MFs), DEGs were significantly involved in extracellular matrix structural constituent (26 genes), collagen binding (10 genes), integrin binding (11 genes), extracellular matrix structural constituent conferring tensile strength (6 genes), and extracellular matrix structural constituent conferring compression resistance (5 genes). Additionally, the enrichment results of the KEGG pathway analysis showed that DEGs were related to phagosomes (8 genes), Staphylococcus aureus infection (6 genes), and protein digestion and absorption (6 genes) (Figure 4C). The top 5 pathways are listed in Table 1.

**Table 1:** TOP 5 KEGG pathway of differentially expressed genes (DEGs).

**ID**	**Description**	**Gene count**	**P value**	**Genes**
hsa03040	Spliceosome	23	2.08×10-9	HNRNPM/SART1/DDX42/HNRNPA3/ISY1/SF3B2/SNRPD3/PUF60/SRSF4/PLRG1/HNRNPA1/CCDC12/SYF2/RNU4-1/WBP11/THOC3/RNU5A-1/LSM3/SNRPA1/SRSF5/RNU2-1/SNRPA/RNU4-2
hsa05014	Amyotrophic lateral sclerosis	32	5.63×10-6	ATXN2/PSMC5/NUP214/NDUFS6/ATF4/PPP3R1/COX5B/HNRNPA3/PSMC1/COX4I1/ATP5MC1/NDUFC2/HNRNPA1/PSMD6/NDUFA12/NDUFA4/PFN1/NDUFA2/PSMA5/RAB1A/NDUFA5/GPX7/PSMA4/COX8A/PSMB3/ATP5F1A/PSMA2/NUP107/COX7A2/SIGMAR1/ACTR1B/COX7B
hsa05014	Prion disease	26	5.04×10-6	PSMC5/NDUFS6/ATF4/PPP3R1/COX5B/PSMC1/COX4I1/ATP5MC1/NDUFC2/PSMD6/NDUFA12/NDUFA4/NDUFA2/PSMA5/NDUFA5/CREB3L2/PSMA4/COX8A/PSMB3/ATP5F1A/PSMA2/CSNK2A2/COX7A2/COX7B/CYBA/NCF2
hsa05014	Oxidative phosphorylation	17	2.25×10-6	NDUFS6/COX5B/COX4I1/ATP5MC1/NDUFC2/NDUFA12/NDUFA4/NDUFA2/NDUFA5/COX8A/ATP5F1A/ATP6V0E1/COX7A2/ATP6V0B/ATP6V1F/COX7B/ATP6V1C1
hsa05012	Parkinson disease	24	2.91×10-5	PSMC5/GNAI3/NDUFS6/ATF4/COX5B/PSMC1/COX4I1/ATP5MC1/NDUFC2/SLC39A7/PSMD6/NDUFA12/NDUFA4/NDUFA2/PSMA5/NDUFA5/PSMA4/COX8A/PSMB3/ATP5F1A/PSMA2/COX7A2/TXN/COX7B

**Figure 5:**
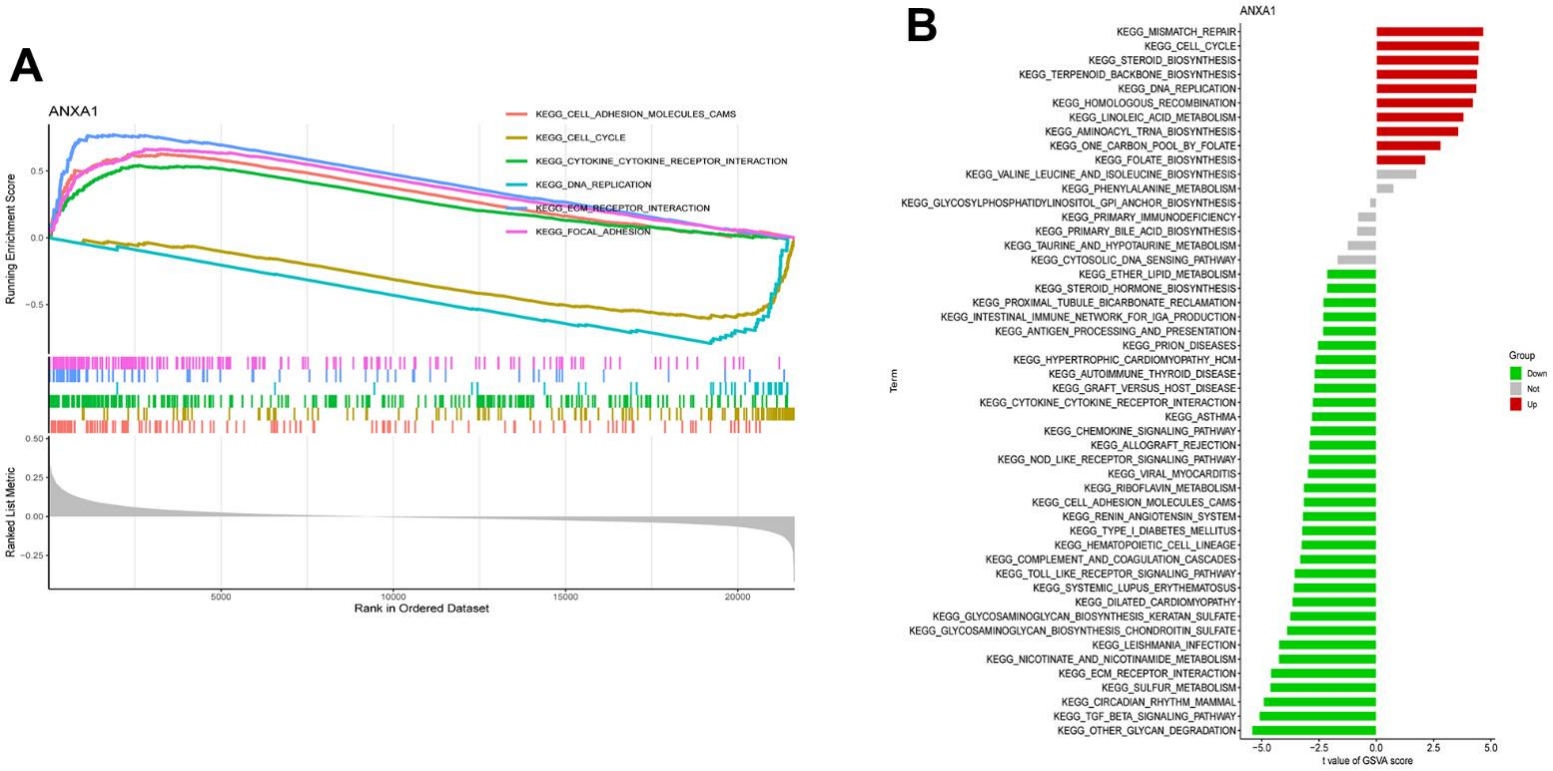
**Single-gene gene set enrichment analysis (ssGSEA) and gene set variation analysis (ssGSVA) pathway analysis of ANXA1.** (**A**) The top 6 underlying related KEGG enrichment pathways of ANXA1 through single-gene GSEA. (**B**) Results of single-gene GSVA analysis involving KEGG pathways.

To further identify the expression differences in the involved KEGG pathways, GSEA and GSVA enrichment analyses revealed that ANXA1 was involved in the cell adhesion molecules cams, cell cycle, cytokine-cytokine receptor interaction, DNA replication, ECM receptor interaction and focal adhesion pathways (Figure 5A, 5B).

### PPI network construction and modular analysis

**Figure 6:**
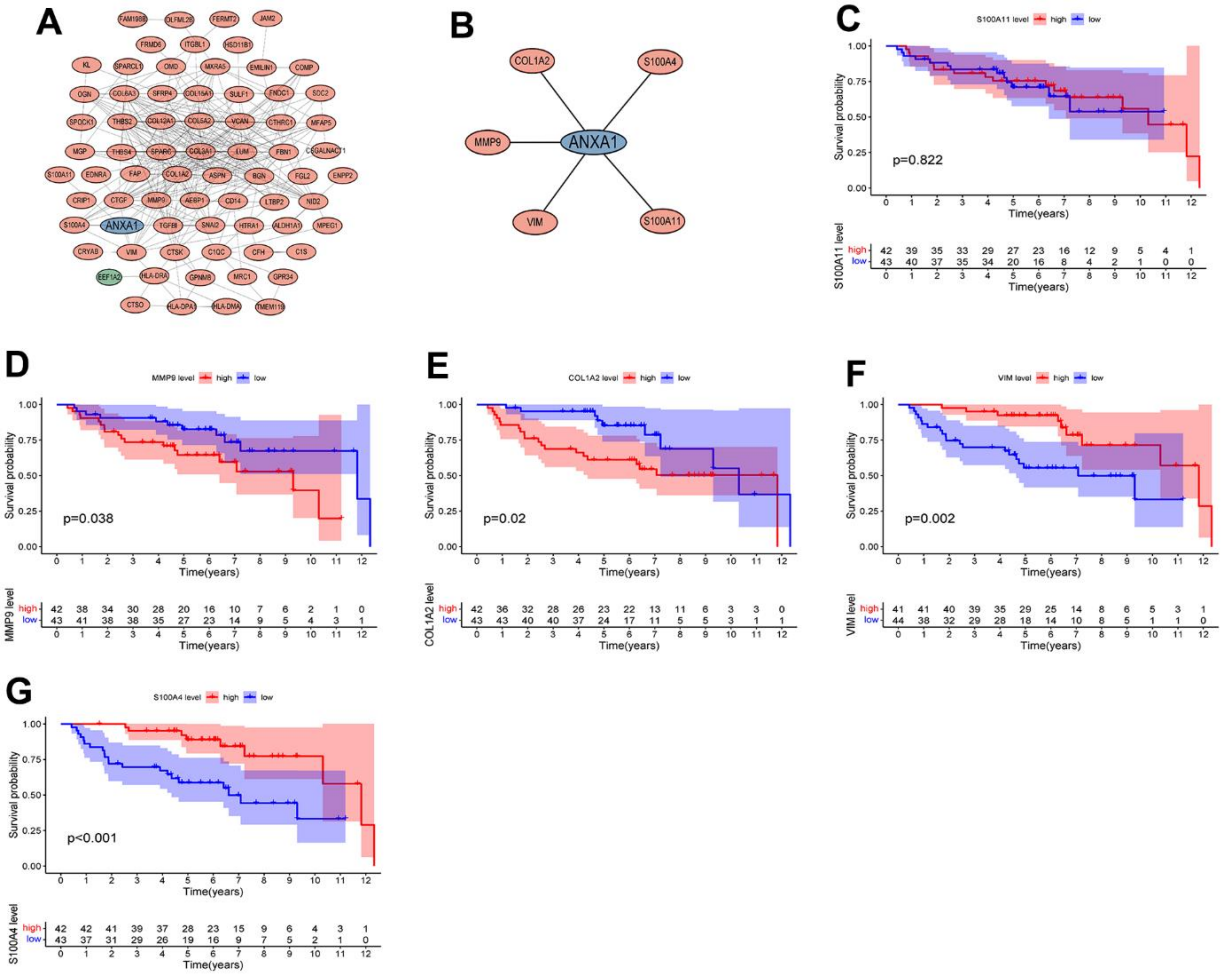
**Protein interaction network diagram and survival curves of module genes.** (**A**) The module with the highest MCODE score. (**B**) The module associated with AXNA1. Red indicates upregulated genes, green indicates downregulated genes, and hub gene is indicated in blue. The expressions of ANXA1-related module genes used to construct the prognosis of ES. (**C**) S100A11; (**D**) MMP9; (**E**) COL1A2; (**F**) VIM; (**G**) S100A4. *P* values <0.05 indicated that differences were significantly significant.

The DEGs were introduced into STRING to draw the PPI network plot. After Cytoscape processing, the module with the highest MCODE score (score=13.875) was constructed (Figure 6A), which included 71 nodes and 304 edges, as well as 69 upregulated genes and 1 downregulated gene. Next, a new module was constructed using ANXA1 as the node, which included COL1A2, MMP9, VIM, S100A11 and S100A4, all of which were upregulated genes (Figure 6B).

### Survival analysis of DEGs between the two groups

The five modular genes were differentially expressed in RD-ES cells or A673 cells compared to MSCs, and we further established survival curves to explore the effects of these genes on the prognosis of ES. The results revealed that except for S100A11 (Figure 6C), the expression of COL1A2, MMP9, VIM and S100A4 significantly affected the overall survival time of patients with ES, in which the OS time of the high expression group of MMP9 and COL1A2 was significantly lower than that of the low expression group (Figure 6D, 6E), and the OS time of the low expression group of VIM and S100A4 was significantly lower than that of the high expression group (Figure 6F, 6G).

### Immune infiltration analysis of ANXA1 and five module genes

**Figure 7:**
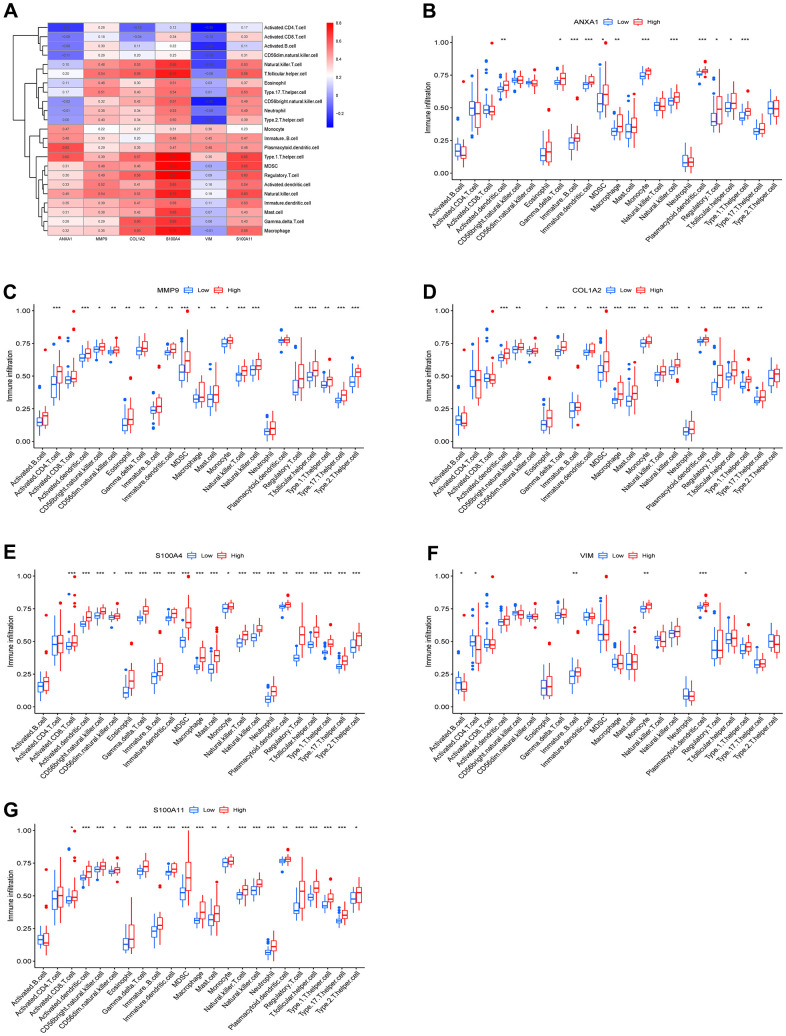
**Immune infiltration results of the 6 hub genes.** (**A**) Heatmap of the correlation between immunocyte abundance and 6 hub genes. Box diagram of immunocyte abundance in the ANXA1 (**B**), MMP9 (**C**), COL1A2 (**D**), S100A4 (**E**), VIM (**F**) and S100A11 (**G**) high- and low-expression groups.

To explore the relationship between the 23 immune cells and the hub genes, a correlation heatmap was constructed (Figure 7A) and it suggested that 5 target genes other than the VIM gene had a strong correlation with 23 immune cells. Specifically, ANXA1, COL1A2, MMP9, S100A4 and S100A11 high-expression groups were associated with high infiltration of activated dendritic cells, gamma delta T cells, immature B cells, immature dendritic cells, MDSCs, macrophages, natural killer cells, regulatory T cells, T follicular helper cells, and type 1 T helper cells (Figure 7B–7G).

**Figure 8:**
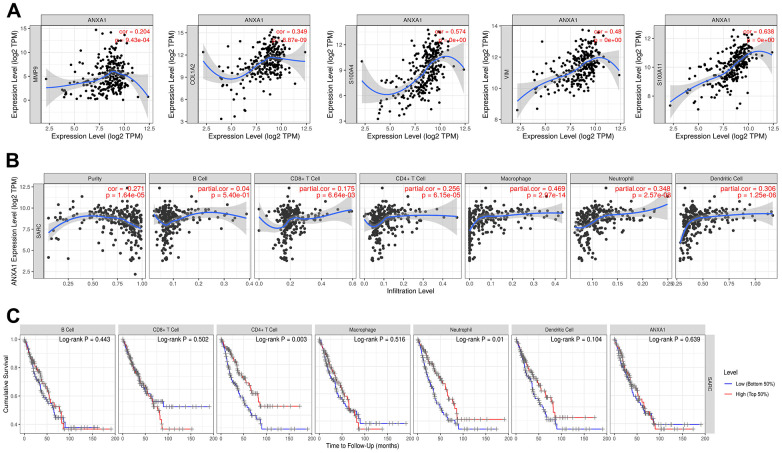
**ANXA1 expression is correlated with 5 module genes and the level of immune infiltration in sarcoma.** (**A**) ANXA1 expression is correlated with 5 module genes in sarcoma. (**B**) ANXA1 expression is correlated with the level of immune infiltration in sarcoma. (**C**) Kaplan–Meier plots of immune infiltration and ANXA1 expression levels in sarcoma.

Previous studies have shown that tumor tissues contain tumor cells and lymphocytes, and TILs can predict the survival status of tumor patients [17, 18]. To further investigate the correlation between the expression levels of ANXA1 and TILs, we explored the correlation between the expression levels of ANXA1 and the infiltrating abundance of TILs in sarcoma patients through the TIMER database. In sarcoma, the expression levels of ANXA1 were positively correlated with those of MMP9 (*r*=0.204, *P*=9.43e-04), COL1A2 (*r*=0.349, *p*=8.87e-09), S100A4 (*r*=0.574, *P*<0.001), VIM (*r*=0.48, *P*<0.001) and S100A11 (*r*=0.638, *P*<0.001) (Figure 8A).

The expression levels of ANXA1 were negatively correlated with tumor purity (*r*=-0.271, *P*=1.64e-05), but positively correlated with the levels of infiltrating B cells (*r*=0.04, *P*=5.4e-01), CD8+ T cells (*r*=0.175, *P*=6.64e-03), CD4+ T cells (*r*=0.256, *P*=6.15e-05), macrophages (*r*=0. 469, *P*=2.97e-14), neutrophils (*r*=0.348, *P*=2.57e-08) and dendritic cells (*r*=0.306, *P*=1.25e-06), with the strongest correlation with macrophages (Figure 8B). We then analyzed the correlation between the expression levels of ANXA1 and immune cells and the prognosis of sarcoma patients, and the Kaplan–Meier plots suggested that the expression levels of CD4+ T cells (*P*=0.003) and neutrophils (*P*=0.01) were significantly associated with the prognosis of sarcoma patients (Figure 8C).

**Figure 9:**
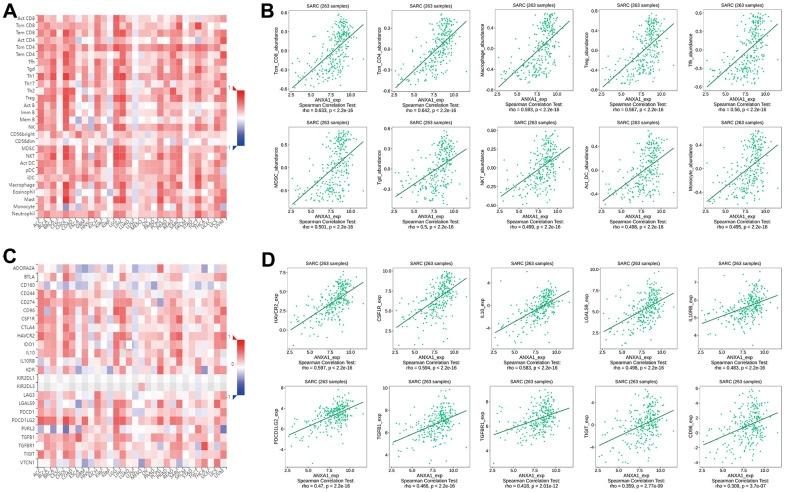
**Correlation of ANXA1 expression with immune cells and immunoinhibitors in sarcoma.** (**A**) Heatmap of the correlation between ANXA1 expression and TILs in sarcoma: red represents a positive correlation, blue represents a negative correlation. (**B**) ANXA1 expression was positively correlated with infiltrating abundance of tcm_CD8, tcm_CD4, macrophage, treg, tfh, MDSC, tgd, NKT, act_DC and monocyte. (**C**) Heatmap of the correlation between ANXA1 expression and immunoinhibitors in sarcoma: red represents positive correlation, blue represents negative correlation. (**D**) ANXA1 expression was positively correlated with infiltrating abundance of HAVCR2, CSF1R, IL-10, LGALS9, IL10RB, PDCD1LG2, TGFB1, TGFBR1, TIGIT and CD96.

Next, we explored the correlation between the expression levels of ANXA1 and immune cells through the TISIDB database. Figure 9A shows a heatmap of correlation between the expression levels of ANXA1 and TILs in different types of tumors. In 263 sarcoma patients, the expression levels of ANXA1 were significantly correlated with the infiltration abundance of 26 TILs, with the strongest correlation with tcm_CD8, tcm_CD4, macrophage, treg, tfh, MDSC, tgd, NKT, act_DC and monocyte (Figure 9B and Supplementary Figure 2).

Finally, we revealed the correlation between the expression levels of ANXA1 and immunoinhibitors in sarcoma patients by a heatmap of correlation (Figure 9C), and the results suggested that the expression levels of ANXA1 were associated with 20 immunoinhibitors, among which HAVCR2, CSF1R, IL-10, LGALS9, IL10RB, PDCD1LG2, TGFB1, TGFBR1, TIGIT and CD96 showed strong positive correlations (Figure 9D and Supplementary Figure 3). These results suggested that ANXA1 may play an important role in the immunotherapy of sarcoma.

### The expression levels of ANXA1 and five module genes

**Figure 10:**
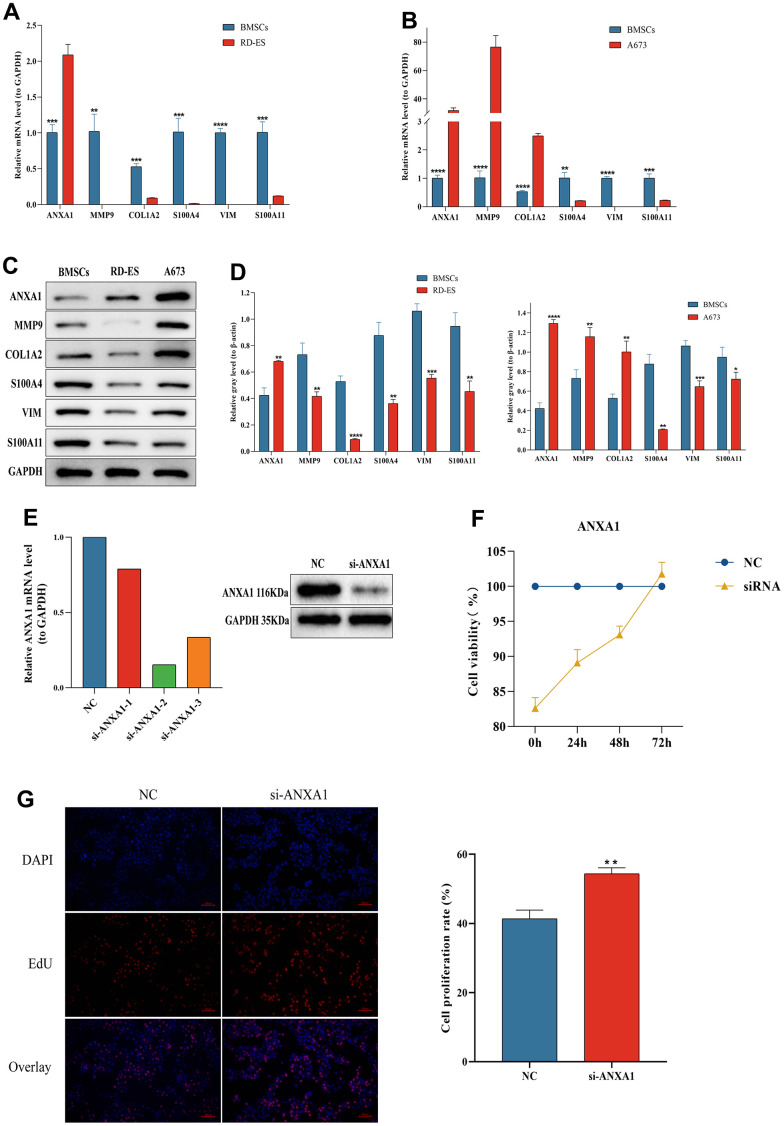
**The expression levels of ANXA1-related module genes in ES cell lines and MSCs by RT-qPCR and Western blot and functional validation of ANXA1 in RD-ES cell using CCK-8 and EdU assays.** The relative mRNA levels of ANXA1, MMP9, COL1A2, S100A4, VIM and S100A11 in RD-ES (**A**) or A673 (**B**) cells compared to MSCs. (**C**) The protein levels of ANXA1-related module genes in ES cell lines and MSCs by Western blot. (**D**) Quantitative analysis of protein expression levels of module genes. (**E**) The efficiency of si-ANXA1 was detected by RT-qPCR and Western blot analysis. (**F**) ES-RD cell viability at 24 h, 48 h and 72 h after downregulation of ANXA1 was measured using a CCK-8 assay. (**G**) The effect of ANXA1 on ES-RD cell proliferation was assessed by an EdU assay. *P* values are shown as follows: * *P*<0.05, ** *P*<0.01, *** *P*<0.001, and **** *P*<0.0001.

The RT-qPCR results showed that six hub genes, including ANXA1, were significantly differentially expressed in either RD-ES cells or A673 cells (Figure 10A). The relative expression levels of ANXA1 were significantly higher in RD-ES cells than in MSCs, while the relative expression levels of MMP9, COL1A2, S100A4, VIM and S100A11 were significantly lower than in MSCs. The relative expression levels of ANXA1, MMP9 and COL1A2 were significantly higher in A673 cells, while the relative expression levels of S100A4, VIM and S100A11 were significantly lower than those in MSCs (Figure 10B). Subsequent Western blot analysis verified that the protein levels of the six hub genes were all significantly differentially expressed in either RD-ES cells or A673 cells (Figure 10C). The expression of ANXA1 increased in ED-ES and A673 cells, while the expression of MMP9, COL1A2, S100A4, VIM and S100A11 decreased in ED-ES cells. It is worth mentioning that the protein levels of ANXA1, MMP9 and COL1A2 were significantly increased in A673 cells, while the protein levels of S100A4, VIM and S100A11 were significantly decreased in A673 cells, which was consistent with the results of RT-qPCR (Figure 10D).

### ANXA1 inhibits the proliferation of RD-ES cells *in vitro*

Since ANXA1 downregulation was associated with worse prognosis and metastasis in ES patients, we further investigated the effect of ANXA1 downregulation on RD-ES cells *in vitro*. Figure 10E showed the efficiency test of si-ANXA1. CCK-8 and EdU assays revealed that, compared with NC group, the proliferation ability of cells in the HMGB1 knockdown group was increased (Figure 10F, 10G). The above results suggested that downregulated ANXA1 may affect the prognosis and metastasis of ES patients by promoting cell proliferation.

## DISCUSSION

As a common malignancy of bone and soft tissue, the prognosis is not promising for the pediatric and adolescent population, even after standardized chemotherapy regimens [19, 20]. With the development of genomics and high-throughput technologies providing a novel direction for the prognosis and treatment of ES, it is crucial to explore key biomarkers for the prognosis of ES patients. In this study, we first screened the ANXA1 gene using independent prognostic analysis and clinical correlation analysis from 88 ES samples obtained from the GSE17679 dataset. Then, survival analysis and clinical correlation analysis based on ANXA1 determined that low ANXA1 expression was associated with a shorter survival time, and this result was successfully validated by 85 ES samples from the GSE63157 dataset. In the clinical subgroups, the expression levels of ANXA1 were significantly lower in the metastatic group than in the primary group. We further analyzed 96 DEGs with ANXA1 expression as the median value and GO and KEGG pathway analyses suggested that DEGs were mainly associated with the extracellular matrix. Finally, we found that the expression of five ANXA1-related modular genes was significantly different between ES cell lines and MSCs by *in vitro* RT-qPCR, and four genes were significantly associated with the prognosis of ES patients.

ANXA1 is a phospholipid-binding protein located on chromosome 9q12-q21.2 and is expressed in many tissues and cells [21, 22]. Its C-terminus is composed of four annexin repeats with calcium binding sites, while the N-terminus contains important phosphorylation regulatory sites, which are unique to ANXA1 [23, 24]. In addition to mediating inflammatory responses, ANXA1 is involved in the development of multiple tumors, metastasis and drug resistance and may be used as a potential biomarker for tumor diagnosis, treatment, and prognosis [25, 26]. However, the role of ANXA1 in tumors remains uncertain, both as an antitumor factor and as a promoter of tumor progression [27]. To date, there is no literature on the relationship between ANXA1 expression and the prognosis of ES. It shows high expression levels in breast [28], colorectal [29] and prostate cancers [30]. This is consistent with the results of the present study, in which we found that ANXA1 showed significantly high expression in either RD-ES or A673 cells and a significant correlation with the OS time of patients with ES. In contrast, ANXA1 shows low expression levels in thyroid cancer [31] and nasopharyngeal carcinoma [32]. A recent study showed that ANXA1 expression was upregulated in patients with small cell lung cancer bone metastases and promoted bone metastasis of small cell lung cancer cells in mice, suggesting that ANXA1 may be a potential biomarker for lung cancer bone metastasis [33]. Interestingly, low expression levels of ANXA1 in this study were significantly associated with ES metastasis, suggesting that the downregulated ANXA1 gene was associated with the proliferation, invasion, and migration of ES cells.

The tumor microenvironment (TME) plays a key role in tumor and developmental processes and therapeutic susceptibility, and the typical immune profile of ES is a large infiltration of myeloid cells in the TME [34, 35]. Myeloid-derived suppressor cells (MDSCs) are involved in a variety of immunosuppressive responses as immature monocytes and granulocytes, and Zhang et al. [36] identified a subpopulation of MDSCs with significant immunosuppressive effects. Patients with ES showed significant depletion of CD4+ and CD8+ T cells in the peripheral blood compared to the healthy population [37]. Consistent with these results, ANXA1 downregulation in this study was significantly associated with reduced infiltration of macrophages, MDSCs and T cells, suggesting that ANXA1 is involved in the regulation of immune responses in the TME of ES patients. Previous studies showed that ANXA1 was involved in the proliferation and invasion of a variety of tumor cells [38, 39], and we also found that downregulated ANXA1 promoted the proliferation of ES cells, which may lead to progression and metastasis in ES patients.

Additionally, the COL1A2 gene was shown to be upregulated in ES tissues [40], and we also obtained consistent results in RD-ES cells. Zhang et al. [41] found that COL1A2 was involved in the development of ES and was significantly associated with the survival time of ES patients. Our survival curves also suggest that patients with low COL1A2 expression have shorter survival times. The S100A11 gene was found to be significantly upregulated in clear cell sarcoma of soft tissue [42]. In contrast, we found higher levels of S100A11 expression in ES-RD and A673 cells. High expression of MMP9 is associated with human tumor invasion or metastasis, and knockdown of MMP inhibits the migration of ES cells [43]. In this study, we verified the relationship between the expression of six key genes and ES and found that five of them were significantly associated with the prognosis of ES. At present, there are no literature reports of MMP9, S100A11, VIM and S100A4 as prognostic markers for ES, and their prognostic relationships need to be further investigated.

In this study, to screen for the hub gene with prognostic significance in ES patients, DEGs were screened using survival analysis and Cox regression analysis based on the OS time and status of ES patients. To reflect the clinical application value of hub genes, patients were further grouped according to their age, gender, and tumor status to explore the hub gene significantly associated with clinical characteristics and prognosis. Finally, differences in the gene and protein expression levels of the hub gene were examined in different ES cell lines and BMSCs using independent datasets to validate its prognostic value, which may provide theoretical support for prognosis and molecularly targeted therapy in clinical ES patients.

Admittedly, our study has some limitations that need to be considered. Firstly, both datasets used for analysis were small and a larger chip set should be utilized to validate the prognostic role of ANXA1 in ES. Secondly, the GEO dataset of the validation cohort lacked detailed clinical information about the patients, making it difficult to validate the relationship between ANXA1 and the clinical characteristics of ES patients. Finally, some basic experiments are also required to further investigate the effect of ANXA1 on the phenotypes of ES cells such as migration.

## CONCLUSIONS

Taken together, we screened ANXA1 as an independent prognostic gene for ES based on the GEO database using multifactorial Cox regression analysis. ES patients with low expression of this gene had a shorter survival time and were significantly associated with ES metastasis and immunosuppressive microenvironment. In addition, the expression levels of ANXA1 were higher in ES cell lines. This study may facilitate the development of new prognostic biomarkers for targeted treatment of ES, although further experimental validations are needed.

## MATERIALS AND METHODS

### Collection and processing of GEO data

Data on ES patients and clinical characteristics were obtained through the Gene Expression Omnibus website (https://www.ncbi.nlm.nih.gov/geo/). Four microarray datasets, GSE17679, GSE63157, GSE45544 and GSE73166, were extracted and the platform files of both datasets were downloaded. 88 ES samples and 18 normal samples from the GSE17679 dataset were obtained from the GPL570 platform, 85 ES samples from the GSE63157 dataset were obtained from the GPL5175 platform, and a total of 22 ES samples from the GSE45544 and GSE73166 datasets were obtained from GPL6244. To extract the survival time and survival status of ES patients, non-ES samples and ES cell lines were excluded. The GSE17679 dataset was the experimental group and the GSE63157, GSE45544 and GSE73166 datasets were the validation group. The results of survival analysis were validated by the GSE63157 dataset, and the results of clinical correlation analysis were validated by the GSE45544 and GSE73166 datasets after eliminating the batch effect.

### Screening of hub gene related to survival and prognosis

First, the data from the gene expression matrix were corrected using the “impute” package of R software (version 4.1.3) [44]. For data with large values of gene expression, log 2 processing is needed. Next, the survival time and survival status of patients were extracted, and the gene expression data and survival information were merged using the “Perl” program [45]. The merged data were subjected to Cox regression analysis to filter genes according to overall survival (OS) time and survival status with a *P* value < 0.001. After obtaining the genes, survival analysis was performed through the “survival” package to screen for survival-associated genes with a *P* value threshold of 0.001. Finally, the samples were divided into two subgroups based on clinical characteristics such as sex and age, and three subgroups based on tumor status. Further clinical correlation analysis of genes was performed to identify the hub gene with a significant number (SigNum) of differences.

### Survival analysis of hub gene and validation

Based on the median expression of the hub gene, 88 ES samples were divided into two groups for survival analysis. Furthermore, survival analyses for patients with different types of ES were performed. The high expression group indicated that the expression of the hub gene was greater than the median, and the low expression group indicated that the expression was less than or equal to the median. Moreover, 85 ES samples from the GSE63157 dataset were used to verify the accuracy of the survival curve.

### Univariate and multivariate Cox regression analysis of hub gene

The hub gene expression data, clinical characteristics, and survival data of 88 ES samples were integrated, and univariate and multivariate Cox regression analyses were conducted using the “survival” package to identify characteristics that independently guided ES prognosis and to validate the results with the GSE63157 dataset.

### Clinical correlation analysis of hub gene

ES patients were divided into two subgroups by age using 20 years as the threshold, two subgroups by gender, and three subgroups by tumor status: primary, recurrent, and metastatic. The hub gene was analyzed for differences among the subgroups.

### Identification and correlation analysis of DEGs

Based on the median expression level of the hub gene, ES samples were divided into high and low expression groups. DEGs were screened using the “limma” package, and the concentrated area of upregulated or downregulated genes was indicated by a volcano plot and heatmap. The screening criteria were an absolute value of |logFC| > 1 and an adjusted *P* value < 0.05. To explore the relationship between the hub gene and DEGs, a significant *P* value was obtained by correlation test. Further visualization was performed in the form of a heatmap and correlation coefficient heatmap using the “corrplot” package.

### GO and KEGG enrichment analysis

Gene Oncology (GO) and Kyoto Encyclopedia of Genes and Genomes Pathway Enrichment (KEGG) enrichment analyses based on DEGs were conducted using the “ClusterProfile” and “enrichplot” packages [46, 47]. GO enrichment analysis focused on the cytological components (CC), molecular functions (MF), and biological processes (BP) of DEGs, while KEGG enrichment analysis focused on the pathways of DEGs. The filtering condition was set to a *P* value < 0.05.

### Single-gene gene set enrichment analysis (GSEA) enrichment

To further analyze the related pathways and potential biological functions of the hub gene in osteomyelitis, we used the “enrichplot” and “clusterProfiler” packages to perform GSEA enrichment analysis for each signature gene, with two gene sets, “c5.go.symbols.gmt” and “c2.cp.kegg.symbols.gmt”, as the predefined sets. The top 6 pathways with significant enrichment were visualized and the screening threshold was set at a *P* value < 0.05.

### Single-gene gene set variation analysis (GSVA) enrichment

GSVA is a method to estimate the variation in pathway activity in samples with an unsupervised way due to its stability and is often used in the data analysis of gene expression profiles [48]. The hub gene was analyzed by GSVA based on predefined sets “c5.go.symbols.gmt” and “c2.cp.kegg.symbols.gmt”. Firstly, the samples are scored and corrected. Then, the samples were divided into groups according to the expression of the target gene, and the difference in GSVA score between the high expression group and the low expression group was further analyzed. The screening conditions for significant differences were |t| >2, *P* value < 0.05.

### Construction of the PPI network and module analysis

The protein-protein interaction network of the hub genes was constructed using STRING (http://string-db.org) [49]. Cluster analysis was further performed using the Molecular Complex Detection (MCODE) plugin in Cytoscape (version 3.9.1) [50] to identify key modules in the PPI network to construct subnetworks.

### Immune infiltration analysis and survival analysis of module genes

We further used ssGSEA in the “gsva” package to combine the 23 immune gene datasets with “high-low discriminant analysis” and calculate the immune infiltration score for each ES sample [51]. The correlation between hub genes and immune cell infiltration was represented by a heatmap. Based on the median expression level of each gene, the samples were divided into low and high expression groups, and boxplots were used to observe whether there was a significant difference in the abundance of immune cells between the high and low expression groups. Based on the median expression values of the hub gene, survival analysis was divided into high and low expression groups.

### TIMER database

The TIMER database serves as an online visualization tool to assess the correlation between immune cell infiltration and target genes in different cancers (https://cistrome.shinyapps.io/timer/) [52]. In the current study, we analyzed the correlations between the infiltration abundance of B cells, CD8+ T cells, CD4+ T cells, macrophage, neutrophil and dendritic cells and expression levels of ANXA1 in sarcoma by TIMER database.

### TISIDB database

The TISIDB database is an integrated repository portal for tumor-immune system interactions (http://cis.hku.hk/TISIDB/) [53]. Correlations between the infiltration abundance of tumor infiltrating lymphocytes (TILs) and the expression levels of ANXA1 in sarcoma evaluated by the TISIDB database.

### Cell culture

ES is a malignant bone tumor that invades bone or muscle tissue, and mesenchymal stem cells are its main precursor cells [54]. Therefore, we selected RD-ES and A673 cells (Procell Bio, Wuhan, China) as the experimental group and MSCs as the control group for the experiment. Cells were maintained in Dulbecco’s modified Eagle’s medium (Gibco, NY, USA) supplemented with 10% fetal bovine serum (Gibco, NY, USA), 100 U/mL penicillin and 100 mg/mL streptomycin. Cultures were incubated at 37° C and 5% CO2.

### RNA interference

Specific small interfering RNAs (siRNAs) were obtained from Guangzhou RiboBio Co., Ltd. ANXA1 siRNA or negative control (NC) siRNA was transfected into cells. The siRNA-ANXA1 sequences were as follows: #1, 5′-CAUAAGGCCAUAAUGGUUAAATT-3′; #2, 5′-UUUAACCAUUAUGGCCUUAUGTT-3′; #3, 5′-GCAUUCUAUCAGAAGAUGUAUTT-3′.

### Cell survival test (CCK-8 assay)

Cells were inoculated into 96-well plates and cultured overnight, followed by transfection of cells with si-ANXA1 and si-NC. Ten microliters of CCK-8 were added to each well, and all experimental procedures were performed according to the instructions of the CCK-8 kit (Sigma-Aldrich, St. Louis, MO, USA).

### Cell proliferation test (EdU assay)

Cells were inoculated into 24-well plates, and EdU reagent was added to each well at a ratio of 1:1000. The cells were fixed and stained according to the instructions of the EdU Cell Proliferation Kit (Beyotimebio, Shanghai, China), followed by observation of the cells using a fluorescence microscope.

### RNA extraction and real-time quantitative PCR

The TRIzol (Ambion LLC, Austin, TX, USA) was used to extract total RNA from cells, which was reverse transcribed into cDNA using a reverse transcription kit (Service Bio, Guangzhou, China). Real-time quantitative PCR was performed using Universal Blue SYBR Green qPCR Master Mix (Service Bio, Guangzhou, China). After brief centrifugation, reverse transcription was performed on a general PCR instrument under the following conditions: denaturation at 95° C for 20 s, annealing at 55° C for 20 s, and extension at 72° C for 30 s. The expression levels of GAPDH were used as an internal control. The primer sequences are listed in Table 2.

**Table 2:** The primer used for hub gene and module genes.

**Targeted gene**	**Forward (5′-3′)**	**Reverse (3′-5′)**
ANXA1	CTTTCTCTTGCTAAGGGTGA	TGGTGGTAAGGATGGTATTG
COL1A2	CAAAGGAGAGAGCGGTAACA	GAAGACCACGAGAACCAGGA
S100A4	CCACCTTCCACAAGTACTCG	GCTTCATCTGTCCTTTTCCC
VIM	TGACCGCTTCGCCAACTA	TTCGGCTTCCCCTCTCTG
S100A11	CCCTGATTGCTGTCTTCC	GGGTCCTTCTGGTTCTTT
MMP9	ATGAGCCTCTGGCAGCCCCTGGTCC	GGACCAGGGGCTGCCAGAGGCTCAT
GAPDH	CCCATCACCATCTTCCAGG	CATCACGCCACAGTTTCCC

### Protein extraction and Western blotting analysis

RIPA lysis buffer (Servicebio, Wuhan, China) supplemented with phosphatase inhibitor (Servicebio, Wuhan, China) was used to extract total cellular proteins from cultured cells on ice for 10 min. The above lysates were centrifuged for 15 min at 16,000 rpm to remove cellular debris, and the supernatant containing proteins was collected and separated into 80 μl aliquots. A BCA Protein Assay Kit (Beyotimebio, Shanghai, China) was used to measure the protein concentration in the supernatant, and the separated proteins were transferred to PVDF membranes (Millipore, MA, USA). Subsequently, the membranes were blocked with 5% (w/v) skimmed milk (Servicebio, Wuhan, China) in Tris-buffered saline with Tween 20 (TBST; Solarbio, Beijing, China) for 60 min at 37° C. Primary antibodies were then probed overnight at 4° C in 1% (w/v) skimmed milk in TBST. After three washes with TBST, membranes were incubated with the appropriate horseradish peroxidase (HRP)-linked secondary antibodies for 60 min. Antibody reactivity was detected using the ECL Chemiluminescent substrate (Millipore, MA, USA). Gray values were analyzed using ImageJ software. Blots were representative of 3 independent experiments, with quantified results expressed as the means ± standard deviations (SD). The primary antibodies were as follows: ANXA1 (YT0234; Immunoway, Suzhou, China, 1:1500), MMP9 (YT1892; Immunoway, Suzhou, China, 1:1500), COL1A2 (YM4409; Immunoway, Suzhou, China, 1:1500), S100A4 (YM1458; Immunoway, Suzhou, China, 1:1000), VIM (YT4879; Immunoway, Suzhou, China, 1:1500), S100A11 (10237-1-AP; Proteintech, Wuhan, China, 1:1500), and β-Actain (66009-1-Ig; Proteintech, Wuhan, China, 1:25000).

### Statistical analysis

The external validation of the genes was repeated three times. Statistical analysis was performed using GraphPad Prism (version 8.0), and Student’s t-test was used for comparisons between two groups. Bioinformatics analysis was performed using R 4.1.3.

### Availability of data and materials

The microarray data used to support the findings of this study can be downloaded from the GSE17679, GSE63157, GSE45544 and GSE73166 datasets (https://www.ncbi.nlm.nih.gov/geo). The processed data are available from the corresponding author upon request.

## Supplementary Material

Supplementary Figures

Supplementary Tables 1 and 2

Supplementary Table 3
