# Supplementary material for: Downregulated annexin A1 expression correlates with poor prognosis, metastasis, and immunosuppressive microenvironment in Ewing’s sarcoma
Source: Aging (Albany NY). 2023 Mar 28;15(6):2321–46. doi: 10.18632/aging.204615 (PMC10085606; doi:10.18632/aging.204615)
Supplement: Supplementary Figures [file aging-15-204615-s001.pdf]

SUPPLEMENTARY FIGURES

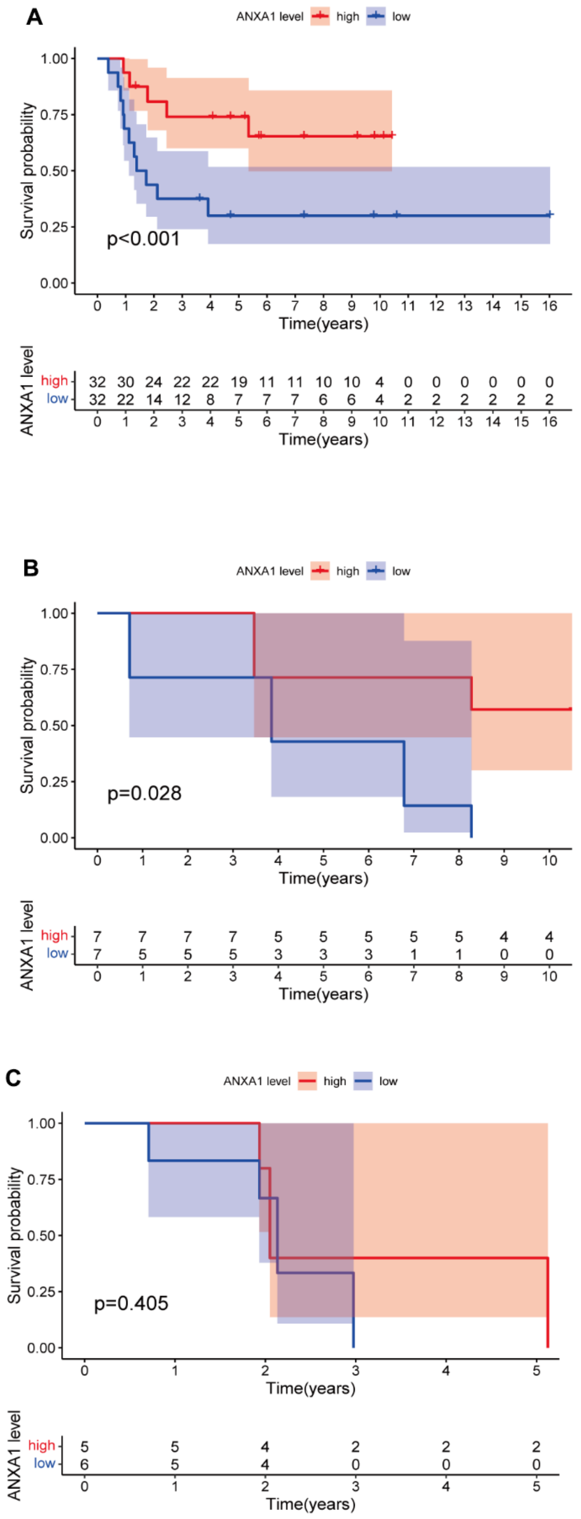

Supplementary Figure 1. The survival analyses were performed in primary ES, recurrent ES, and metastatic ES patients from the GSE17679 dataset. The OS time of ANXA1 low expression group was significantly lower than that of high expression group in primary (**A**) and metastatic ES patients (**B**), while there was no significant difference in recurrent ES patients (**C**).

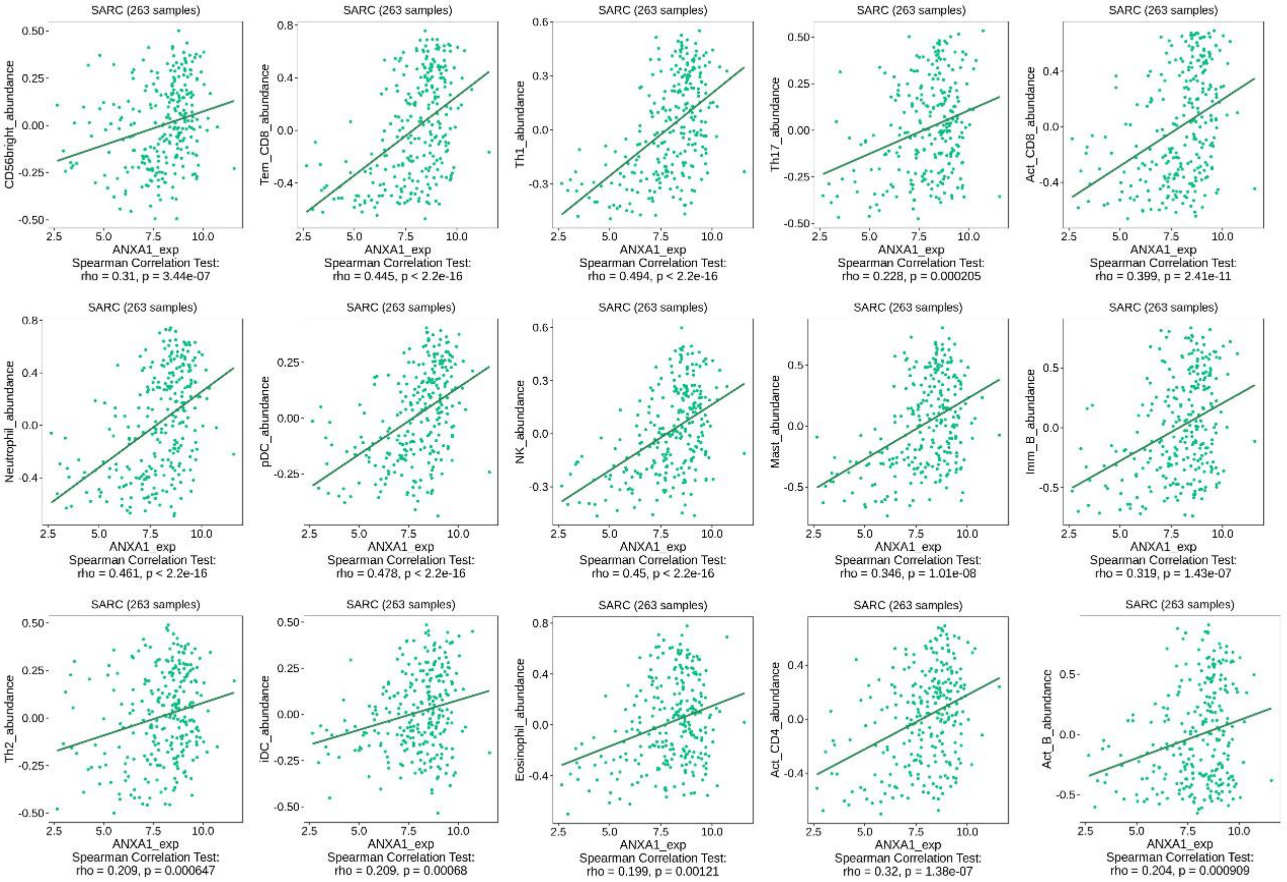

Supplementary Figure 2. Correlation of ANXA1 expression with infiltrating abundance of 15 immune cells in sarcoma.

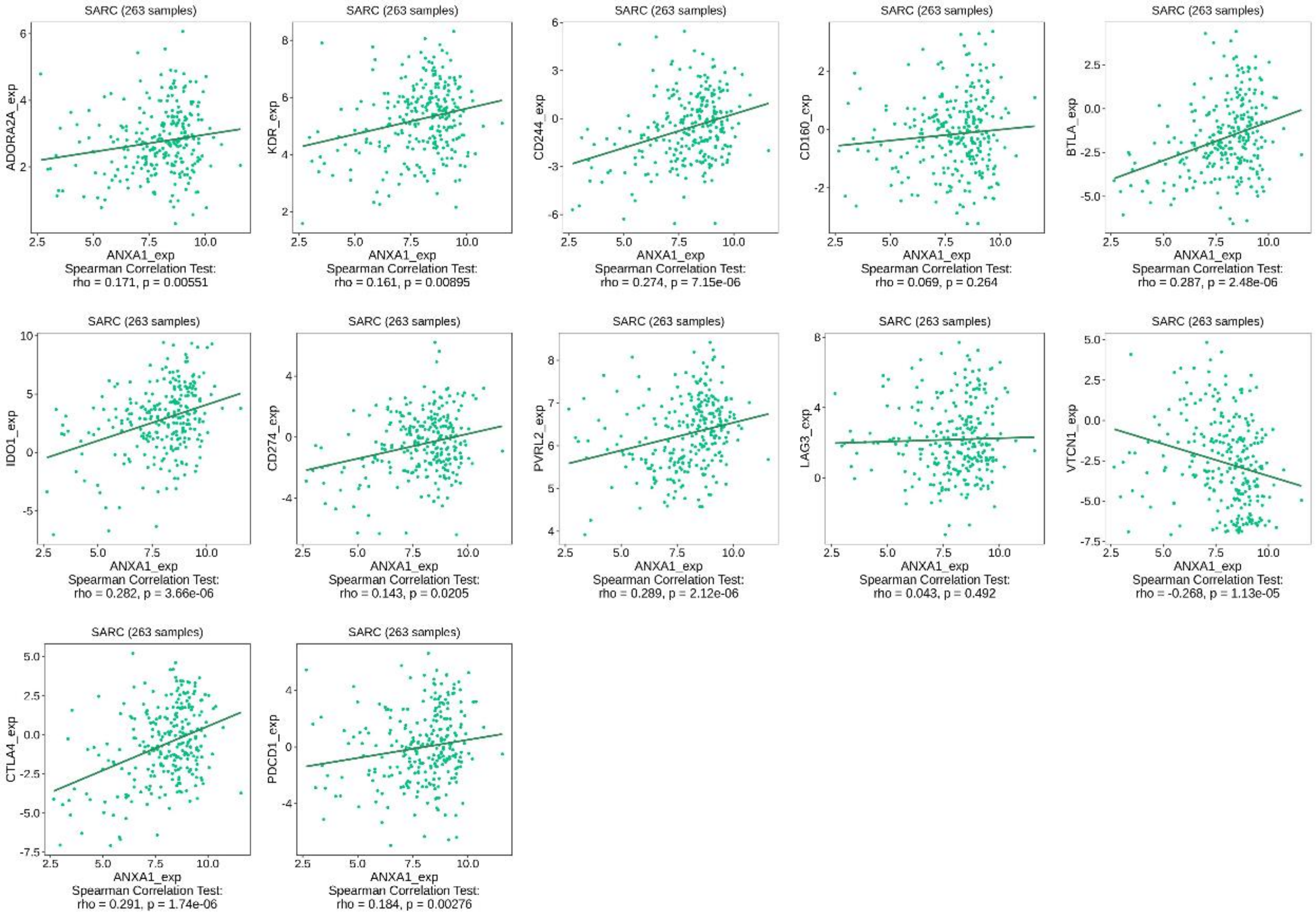

Supplementary Figure 3. Correlation of ANXA1 expression with remained 12 immunoinhibitors in sarcoma.
