# Supplementary material for: Downregulated annexin A1 expression correlates with poor prognosis, metastasis, and immunosuppressive microenvironment in Ewing’s sarcoma
Source: Aging (Albany NY). 2023 Mar 28;15(6):2321–46. doi: 10.18632/aging.204615 (PMC10085606; doi:10.18632/aging.204615)
Supplement: Supplementary Tables 1 and 2 [file aging-15-204615-s002.pdf]

SUPPLEMENTARY TABLES

Supplementary Table 1. The results of 64 genes were screened by Cox regression analysis.

Id	HR	HR.95Low	HR.95High	P value
SSR4P1	0.0946393	0.0253896	0.3527656	0.0004446
BTD	0.1668399	0.0585303	0.475575	0.0008063
CFI	2.784123	1.5546975	4.9857549	0.0005724
ATE1	0.2609063	0.119842	0.5680153	0.0007121
8-Mar	0.2054615	0.0876747	0.4814893	0.0002705
DNAJB12	0.0240698	0.0027312	0.2121219	0.0007894
OR1L1	0.0524297	0.009376	0.2931806	0.0007878
NFASC	5.8062737	2.3898553	14.106634	0.0001029
NUMB	0.1843926	0.0712345	0.4773054	0.0004938
HMGCS1	2.4803271	1.4925393	4.1218495	0.000456
SLC11A2	6.3468759	2.1559216	18.68474	0.0007953
ANXA1	0.4654845	0.3108985	0.6969341	0.0002046
CTSC	1.8600218	1.2947126	2.6721612	0.0007872
CHN1	1.9746884	1.3864601	2.812482	0.0001627
CTPS1	4.8559463	2.1468725	10.983519	0.0001479
INHA	0.2915138	0.1628967	0.5216821	3.30E-05
KCNS3	2.3154706	1.4402569	3.7225332	0.0005283
MAP2	1.7018774	1.241727	2.332547	0.0009461
PSMA7	6.7942523	2.3111685	19.973388	0.0004964
RBMS2	0.2031346	0.0906346	0.4552746	0.0001084
BEST1	4.293332	2.110563	8.733546	5.78E-05
MARS	5.051995	2.2224331	11.484104	0.0001106
SLC20A1	3.3130711	1.6815334	6.5276373	0.0005363
DNAJC4	0.0985114	0.0271812	0.3570295	0.0004192
ATP8B1	0.6241602	0.4780785	0.8148786	0.0005306
NFYB	2.5735752	1.5496411	4.2740796	0.0002598
CHL1	1.5858409	1.2146507	2.0704645	0.0007009
EIF2D	0.0662968	0.0164734	0.2668104	0.0001336
ZHX2	0.2079355	0.085234	0.5072762	0.0005574
YTHDF2	9.4767684	2.5705217	34.938098	0.0007296
KIF21B	2.1792797	1.3965846	3.4006245	0.0006007
PDPR	0.2766747	0.1417277	0.5401123	0.0001667
NETO2	1.9259348	1.4275289	2.5983536	1.79E-05
FAM63A	0.1917146	0.0726714	0.5057627	0.000846
H2AFY2	0.5248026	0.3617387	0.761372	0.0006838
DDX55	10.282556	3.2278459	32.75589	8.07E-05
CLSPN	2.6456275	1.5176065	4.6120946	0.0006013
AUNIP	5.2853782	2.6188816	10.666852	3.37E-06
ADIPOR2	14.145382	3.0340257	65.949288	0.0007436
ACTR5	15.301172	3.2723971	71.545673	0.0005274
KIAA0226L	1.7733205	1.2643501	2.4871794	0.0009037
TET1	0.2975943	0.1634802	0.5417316	7.32E-05
NBR1	0.1905661	0.0787456	0.4611745	0.0002365
FAXDC2	0.224197	0.1146697	0.4383399	1.24E-05

FBXL20	0.0994482	0.035282	0.2803108	1.27E-05
COX15	0.1551205	0.0549719	0.4377209	0.0004301
BRI3BP	7.1090019	2.4195744	20.887107	0.0003614
FGD4	1.9144158	1.3700471	2.6750817	0.0001422
CYB5D2	0.1317974	0.045288	0.3835571	0.0002007
IL20RB	2.0743551	1.4175644	3.0354523	0.0001724
APOBEC3F	0.2262472	0.1001547	0.5110875	0.0003511
CDRT4	0.2739683	0.1355931	0.553558	0.0003087
ZDHHC21	0.5290647	0.3717798	0.7528904	0.0004051
KRTAP12-2	1.6354243	1.2517914	2.1366279	0.0003104
LSMEM1	12.346787	4.5333341	33.627158	8.80E-07
SUMF1	0.036109	0.0058661	0.2222713	0.0003412
SLC6A15	1.6407141	1.3189495	2.0409749	8.77E-06
RPS19BP1	5.8278403	2.0542064	16.533744	0.0009228
E2F7	4.731335	2.2577636	9.9149138	3.83E-05
SERTM1	0.5016163	0.3405438	0.7388738	0.0004804
ZBTB18	6.0914927	2.6841604	13.824168	1.55E-05
ANKRD18DP	0.7070647	0.5772076	0.8661363	0.0008138
FOXP2	2.8732711	1.8082392	4.5655944	7.93E-06
CARS	9.8798473	3.3842589	28.842764	2.79E-05

Supplementary Table 2. The results of 50 genes were further screened by survival analysis.

Gene	KM	HR	HR.95Low	HR.95High	coxPvalue
ANXA1	0.0017564	0.0925683	0.0334393	0.2562527	4.63E-06
FOXP2	0.0128039	2.8935373	1.8359108	4.5604382	4.71E-06
LSMEM1	0.0060463	9.7600715	3.67649	25.91031	4.79E-06
SLC6A15	0.003197	1.6474721	1.3263386	2.0463586	6.39E-06
ZBTB18	0.0096835	6.2167395	2.7463182	14.072605	1.17E-05
AUNIP	0.000659	4.3322096	2.2412261	8.374006	1.30E-05
CARS	0.0365124	10.768978	3.6693034	31.605695	1.52E-05
BEST1	0.0103564	4.3078835	2.204553	8.4179698	1.93E-05
FAXDC2	0.0026379	0.2333351	0.1192799	0.4564497	2.13E-05
TET1	0.0003299	0.3419903	0.2084827	0.5609932	2.15E-05
NETO2	0.0046757	1.8102402	1.3520919	2.4236294	6.72E-05
DDX55	0.0006837	10.72174	3.3290666	34.530912	7.03E-05
MARS	0.0061805	5.019813	2.2498657	11.200012	8.14E-05
INHA	0.0255581	0.3017491	0.1659517	0.5486689	8.58E-05
E2F7	0.011152	4.1732237	2.0446938	8.517557	8.68E-05
NFASC	0.0162706	5.5638996	2.3606329	13.113847	8.73E-05
EIF2D	0.0292688	0.0715743	0.0186022	0.2753909	0.0001252
C5	0.0142821	0.4790135	0.3282701	0.6989792	0.0001348
CTPS1	0.0029741	4.6378642	2.0835264	10.32374	0.0001713
NBR1	0.0044568	0.1883685	0.0785576	0.4516775	0.0001832
8-Mar	0.0082468	0.200569	0.0864332	0.4654218	0.0001835
RBMS2	0.0053486	0.2005834	0.0864395	0.4654551	0.0001836
PDPR	0.0060672	0.2759143	0.1400358	0.5436375	0.0001982
CDRT4	0.0391983	0.2715201	0.1365126	0.5400464	0.0002023
KRTAP12-2	0.0090707	1.6503582	1.2656646	2.1519777	0.0002157
IL20RB	0.0058562	2.0682706	1.4029601	3.0490842	0.0002428
CYB5D2	0.0272408	0.137426	0.0474605	0.3979292	0.0002535
FGD4	0.0049209	1.819467	1.318601	2.5105853	0.0002689
BRI3BP	0.0181313	7.0769188	2.45832	20.372767	0.0002864
SUMF1	0.0046908	0.0354402	0.0056778	0.2212143	0.0003508
CFI	0.0044534	2.8362982	1.5931671	5.0494309	0.0003963
APOBEC3F	0.0084779	0.240844	0.109472	0.5298691	0.0004021
ATE1	0.0023368	0.2528916	0.1177655	0.5430636	0.0004224
COX15	0.0344881	0.1615047	0.0584243	0.4464543	0.0004408
KIF21B	0.0080593	2.1930646	1.4136768	3.4021443	0.0004562
PBLD	0.003586	0.2494869	0.1127873	0.5518685	0.0006091
PSMA7	0.0339508	6.395707	2.2105609	18.504384	0.0006184
NFYB	0.0168299	2.3355333	1.4358686	3.7988962	0.000632
CHN1	0.0007542	1.7888529	1.2810915	2.4978659	0.0006397
H2AFY2	0.0055528	0.5237169	0.3595369	0.7628685	0.0007507
ATP8B1	0.0075006	0.6321832	0.4841666	0.8254506	0.0007533
KCNS3	0.0196467	2.2566179	1.4051509	3.6240409	0.0007592
FAM63A	0.0193894	0.1946105	0.0745663	0.5079135	0.0008257
SSR4P1	0.0057688	0.1350519	0.0416813	0.4375832	0.0008442
KIAA0226L	0.0346281	1.8031518	1.2754683	2.549147	0.0008458
ANKRD18DP	0.0006898	0.7192489	0.5925809	0.872993	0.0008554
BTD	0.0006534	0.1731531	0.0617068	0.4858784	0.0008651
NUMB	0.0328087	0.2323718	0.0984309	0.5485745	0.0008685
ASB9P1	0.0055941	4.1440697	1.7917938	9.5844253	0.0008897
CTSC	0.0012302	1.8596511	1.289374	2.6821561	0.0008997
