# Supplementary material for: Downregulated annexin A1 expression correlates with poor prognosis, metastasis, and immunosuppressive microenvironment in Ewing’s sarcoma
Source: Aging (Albany NY). 2023 Mar 28;15(6):2321–46. doi: 10.18632/aging.204615 (PMC10085606; doi:10.18632/aging.204615)
Supplement: Supplementary Table 3 [file aging-15-204615-s003.docx]

**Supplementary Table 3**. The results of 50 genes were significantly associated with two clinical features, and 324 genes were significantly associated with only one clinical feature through clinical correlation analysis.

| **id** | **Age** | **Gender** | **Type** | **SigNum** |
| --- | --- | --- | --- | --- |
| ANXA1 | 0.017674 | 0.0665677 | 0.015211 | 2 |
| AC004941.5 | 0.0469893 | 0.5758293 | 0.0095659 | 2 |
| ADRB3 | 0.0078455 | 0.6771447 | 0.0262926 | 2 |
| AL928742.12 | 0.0045003 | 0.7713696 | 0.0040379 | 2 |
| ANKRD44-IT1 | 0.0170591 | 0.0006625 | 0.0582994 | 2 |
| ARFIP1 | 0.6333037 | 0.0118856 | 0.0096963 | 2 |
| ATF5 | 0.2801708 | 0.0072604 | 0.0456042 | 2 |
| ATP2B3 | 0.4123834 | 0.0025033 | 0.0156191 | 2 |
| BCL9L | 0.0255099 | 1 | 0.0148159 | 2 |
| BIRC2 | 0.0393855 | 0.0569102 | 0.0144976 | 2 |
| C14orf28 | 0.2398561 | 0.0203228 | 0.0017051 | 2 |
| C17orf96 | 0.7384128 | 0.0446355 | 0.029767 | 2 |
| CACNA1B | 0.4025617 | 0.007846 | 0.0018388 | 2 |
| CACNB3 | 0.0301172 | 0.9654059 | 0.020507 | 2 |
| CCT6A | 0.1057136 | 0.0314577 | 0.0003729 | 2 |
| CECR5 | 0.6118479 | 0.0052914 | 0.0043835 | 2 |
| CTSV | 0.0255099 | 0.6930838 | 0.0073365 | 2 |
| DNAJC2 | 0.4556797 | 0.0191792 | 0.0059095 | 2 |
| DPYSL4 | 0.0843182 | 0.0278847 | 0.0021646 | 2 |
| EIF3F | 0.5967154 | 0.0170596 | 0.0137263 | 2 |
| ELF1 | 0.2840498 | 0.0058085 | 0.0005493 | 2 |
| EXOC1 | 0.5351253 | 0.017674 | 0.0314399 | 2 |
| FABP5 | 0.0246641 | 0.2100511 | 0.0293723 | 2 |
| FAM228B | 0.0109009 | 0.0569135 | 0.0106196 | 2 |
| FGF6 | 0.0191782 | 0.0032305 | 0.8666975 | 2 |
| FLAD1 | 0.5582049 | 0.0174666 | 0.0316847 | 2 |
| GATAD1 | 0.6363981 | 0.0255099 | 0.0257703 | 2 |
| GINS4 | 0.014615 | 0.4349929 | 0.00146 | 2 |
| GLRX5 | 0.7980362 | 0.0354222 | 0.0059906 | 2 |
| KLHL23 | 0.0076464 | 0.3510934 | 0.0004887 | 2 |
| KRI1 | 0.0089158 | 0.2007415 | 0.0138524 | 2 |
| KRT25 | 0.0054348 | 0.0484537 | 0.0538856 | 2 |
| LINC00847 | 0.0354238 | 0.6930838 | 0.0024054 | 2 |
| LINC00959 | 0.6488269 | 0.0180933 | 0.0408347 | 2 |
| LONP1 | 0.6613486 | 0.015889 | 0.0005072 | 2 |
| NANP | 0.0410752 | 0.9412248 | 0.0087701 | 2 |
| NAT8L | 0.0203228 | 0.0160809 | 0.2445682 | 2 |
| NCAPH2 | 0.0365741 | 0.2613052 | 0.0008921 | 2 |
| NCOA4 | 0.1322993 | 0.0160715 | 0.0001192 | 2 |
| PAGE1 | 0.0212797 | 0.1858996 | 0.0047278 | 2 |
| PAK1IP1 | 0.0040342 | 0.1638618 | 0.0302375 | 2 |
| PAM16 | 0.017673 | 0.095823 | 0.0298566 | 2 |
| PHF11 | 0.6708112 | 0.0017143 | 0.0480951 | 2 |
| PRMT2 | 0.0419429 | 0.453066 | 0.0003057 | 2 |
| PRPF19 | 0.6302156 | 0.0499501 | 0.0019069 | 2 |
| PTP4A2 | 0.3101549 | 0.0059638 | 0.0029238 | 2 |
| RITA1 | 0.0474736 | 0.2329869 | 0.0443208 | 2 |
| RP1-202O8.3 | 0.0718776 | 0.0189575 | 0.0106303 | 2 |
| RP11-403P17.3 | 0.0157029 | 0.2364049 | 0.0050422 | 2 |
| RPF2 | 0.0249416 | 0.3786204 | 0.0245355 | 2 |
| ABCA11P | 0.6363981 | 0.3510934 | 0.0454625 | 1 |
| ABCC1 | 0.280173 | 0.5294306 | 1.77E-05 | 1 |
| ACBD6 | 0.7449667 | 0.1391525 | 0.0248748 | 1 |
| ACER3 | 0.5523907 | 0.758133 | 0.0218644 | 1 |
| ACTR5 | 0.0040342 | 0.1917367 | 0.1026698 | 1 |
| ADA | 0.7780128 | 0.474228 | 0.0106307 | 1 |
| ADPRHL1 | 0.4769031 | 0.5758259 | 0.0359614 | 1 |
| AHCTF1 | 0.4198379 | 0.7123854 | 0.0034794 | 1 |
| ALKBH4 | 0.798037 | 0.0415067 | 0.1014051 | 1 |
| AMN | 0.043271 | 0.4850073 | 0.071464 | 1 |
| AMT | 0.7059319 | 0.6676539 | 0.0123492 | 1 |
| ANKRA2 | 0.655077 | 0.0157024 | 0.8967895 | 1 |
| ANKRD26 | 0.1638618 | 0.0361883 | 0.9314123 | 1 |
| ANKRD34A | 0.8520573 | 3.19E-05 | 0.1231997 | 1 |
| ANKRD49 | 0.2576425 | 0.5294288 | 0.0004469 | 1 |
| ANXA5 | 0.1143807 | 0.5669604 | 0.0024498 | 1 |
| ANXA7 | 0.9861586 | 0.0196284 | 0.0869394 | 1 |
| AP4S1 | 0.7713687 | 0.3952863 | 0.0016876 | 1 |
| AP5M1 | 0.4850073 | 0.1174212 | 0.0388488 | 1 |
| APLP1 | 0.5466065 | 0.0164668 | 0.4642543 | 1 |
| APOC2 | 0.1816049 | 0.0469893 | 0.6901691 | 1 |
| ARF3 | 0.546603 | 0.529427 | 0.0050989 | 1 |
| ASPA | 0.9896188 | 0.0103709 | 0.6911694 | 1 |
| ASRGL1 | 0.1438509 | 0.4850073 | 0.005101 | 1 |
| ATAD3A | 0.3444088 | 0.4025576 | 0.0308082 | 1 |
| ATAD3B | 0.0685178 | 0.2245985 | 0.0262416 | 1 |
| ATM | 0.1511173 | 0.9481284 | 0.0031769 | 1 |
| ATP10D | 0.4001263 | 0.5937137 | 0.0135172 | 1 |
| ATP4A | 0.1858996 | 0.7253612 | 0.0019174 | 1 |
| ATP5O | 0.5967171 | 0.0001075 | 0.2635138 | 1 |
| ATP6V0E2 | 0.4959219 | 0.9205407 | 0.0255776 | 1 |
| AURKA | 0.3786267 | 0.8520567 | 0.0116177 | 1 |
| B4GALNT4 | 0.1705225 | 0.8148267 | 0.0099899 | 1 |
| B4GALT5 | 0.8317041 | 0.6488269 | 0.0182609 | 1 |
| BAZ2A | 0.4001222 | 0.69308 | 0.006415 | 1 |
| BBIP1 | 0.9033433 | 0.009988 | 0.0511478 | 1 |
| BC047484 | 0.3977039 | 0.2100552 | 0.0152536 | 1 |
| BCAN | 0.5997322 | 0.1486701 | 0.0107523 | 1 |
| BCL2L2 | 0.8793461 | 0.9826989 | 0.0341671 | 1 |
| BCL7A | 0.7813395 | 0.0782623 | 0.0381841 | 1 |
| BIRC7 | 0.8725095 | 0.0117409 | 0.1756569 | 1 |
| BRCA1 | 0.3122377 | 0.6179462 | 0.0320559 | 1 |
| BUB1B | 0.788006 | 0.4198379 | 0.0001561 | 1 |
| C10orf35 | 0.1691774 | 0.7123878 | 0.0410331 | 1 |
| C14orf80 | 0.0760859 | 0.1414894 | 0.0360995 | 1 |
| C16orf13 | 0.0174671 | 0.0932585 | 0.1511745 | 1 |
| C16orf59 | 0.8656814 | 0.7880069 | 0.0129741 | 1 |
| C16orf62 | 0.3646988 | 0.383344 | 0.0042775 | 1 |
| C17orf89 | 0.8827681 | 0.0746605 | 0.0423626 | 1 |
| C1QTNF7 | 0.9343249 | 0.4556797 | 0.0269914 | 1 |
| C20orf194 | 0.1279309 | 0.0615841 | 0.0011287 | 1 |
| C6orf62 | 0.6333037 | 0.8554582 | 0.0322382 | 1 |
| C7orf50 | 0.1887976 | 0.239852 | 0.0241797 | 1 |
| CASR | 0.3421916 | 0.0423765 | 0.0650257 | 1 |
| CAT | 0.9930791 | 0.0014997 | 0.1700964 | 1 |
| CBR4 | 0.7713696 | 0.2100552 | 0.0460086 | 1 |
| CBS | 0.1174212 | 0.7286159 | 0.0489069 | 1 |
| CCDC42B | 0.1426602 | 0.4099065 | 0.0253011 | 1 |
| CCDC59 | 0.086682 | 0.8999086 | 0.0087815 | 1 |
| CCDC64 | 0.5817619 | 0.1830269 | 0.0029658 | 1 |
| CCDC82 | 0.5817652 | 0.4273793 | 0.0275036 | 1 |
| CCNG2 | 0.6302216 | 0.7647432 | 0.0263355 | 1 |
| CCRN4L | 0.0328529 | 0.4426801 | 0.0830746 | 1 |
| CD320 | 0.2821068 | 0.0263787 | 0.9456054 | 1 |
| CDC20 | 0.9067801 | 0.1114509 | 0.0344691 | 1 |
| CDC25A | 0.4583059 | 0.6994971 | 0.0052598 | 1 |
| CDC25B | 0.2782439 | 0.0151501 | 0.3707594 | 1 |
| CDC45 | 0.5847405 | 0.5408489 | 0.0268162 | 1 |
| CDC7 | 0.3928796 | 0.5877246 | 0.000187 | 1 |
| CDCA2 | 0.5125317 | 0.7027106 | 0.0328836 | 1 |
| CDCA3 | 0.9861586 | 0.7482512 | 0.0010005 | 1 |
| CDK4 | 0.7482469 | 0.3952759 | 0.0120936 | 1 |
| CDKN2A | 0.1134143 | 0.0304491 | 0.1091134 | 1 |
| CENPF | 0.4688871 | 0.3555924 | 0.0062748 | 1 |
| CENPL | 0.5817652 | 0.1038642 | 0.0158738 | 1 |
| CENPM | 0.5758309 | 0.7680533 | 0.0074363 | 1 |
| CENPV | 0.6488269 | 0.7780128 | 0.0058151 | 1 |
| CENPW | 0.383344 | 0.9688636 | 0.00114 | 1 |
| CHTF18 | 0.3040013 | 0.5699271 | 0.0019017 | 1 |
| CKS2 | 0.4662213 | 0.1787816 | 0.0088384 | 1 |
| CLDND2 | 0.0004652 | 0.5817652 | 0.2235705 | 1 |
| CLGN | 0.3786288 | 0.8452612 | 0.0113493 | 1 |
| CNNM1 | 0.2631532 | 0.067863 | 0.0016358 | 1 |
| CNOT2 | 0.061583 | 0.9274299 | 0.0080886 | 1 |
| CNPY2 | 0.0354238 | 0.0514934 | 0.5197955 | 1 |
| COA5 | 0.447858 | 0.326996 | 0.0227899 | 1 |
| COA6 | 0.8384767 | 0.2504224 | 0.0124217 | 1 |
| COX6B1 | 0.2415618 | 0.0233019 | 0.4988015 | 1 |
| CPNE6 | 0.0882921 | 0.0415067 | 0.2878131 | 1 |
| CREB3 | 0.5014251 | 0.5014251 | 0.0048079 | 1 |
| CRK | 0.8930474 | 0.5294306 | 0.0125459 | 1 |
| CSE1L | 0.3762804 | 0.3060441 | 0.0024041 | 1 |
| CSMD1 | 0.6363951 | 0.0494477 | 0.5520981 | 1 |
| CSNK1D | 0.2433386 | 0.0162719 | 0.8056589 | 1 |
| CTD-3126B10.1 | 0.6994959 | 0.0402237 | 0.0799058 | 1 |
| CTPS1 | 0.9067797 | 0.1450439 | 0.0004988 | 1 |
| CUL5 | 0.0441767 | 0.9136568 | 0.83261 | 1 |
| CYFIP2 | 0.6803234 | 0.758134 | 0.0237657 | 1 |
| DDX11 | 0.7780128 | 0.1076046 | 0.0022538 | 1 |
| DENR | 0.3601254 | 0.8418668 | 0.0058299 | 1 |
| DGAT1 | 0.1612498 | 0.7813395 | 0.0005614 | 1 |
| DHRS1 | 0.3510934 | 0.5351253 | 0.0008841 | 1 |
| DHX37 | 0.5209431 | 0.3206134 | 0.0077777 | 1 |
| DMTN | 0.655077 | 0.5041898 | 0.002801 | 1 |
| DNAJC6 | 0.447858 | 0.821568 | 0.000955 | 1 |
| DNM1P35 | 0.3122377 | 0.000776 | 0.8572124 | 1 |
| DNMT1 | 0.1691699 | 0.6930787 | 0.0479724 | 1 |
| DOC2A | 0.3356286 | 0.8896181 | 0.0199641 | 1 |
| DPF1 | 0.44786 | 0.0194038 | 0.6617179 | 1 |
| E2F1 | 0.9965395 | 0.2959147 | 0.0148181 | 1 |
| E2F2 | 0.5640471 | 0.6088025 | 0.0009488 | 1 |
| E2F7 | 0.6994959 | 0.7286159 | 0.0028817 | 1 |
| ECI1 | 0.7913474 | 0.0304491 | 0.4521366 | 1 |
| EEF1A2 | 0.6994959 | 1 | 0.0014988 | 1 |
| EIF4EBP1 | 0.2433406 | 0.3716212 | 0.0107804 | 1 |
| EMILIN1 | 0.5877262 | 0.9896188 | 0.0008792 | 1 |
| ENO2 | 0.6676539 | 0.2038133 | 3.13E-07 | 1 |
| ENPP1 | 0.5523925 | 0.6488269 | 0.0003825 | 1 |
| EP400 | 0.1665018 | 0.2279288 | 0.0114669 | 1 |
| EPB41L4A | 0.1474526 | 0.6333051 | 0.0021084 | 1 |
| ERCC6L | 0.7188632 | 0.5937137 | 0.0082461 | 1 |
| ESCO2 | 0.8588632 | 0.6027477 | 0.0456644 | 1 |
| ESPL1 | 0.7318767 | 0.7846712 | 0.0035478 | 1 |
| EXO1 | 0.9343252 | 0.1076046 | 0.0282688 | 1 |
| FABP3 | 0.6394944 | 0.4123813 | 0.022727 | 1 |
| FAM199X | 0.0185212 | 0.8793466 | 0.9790207 | 1 |
| FAM219A | 0.2959062 | 0.2687383 | 0.0079187 | 1 |
| FAM222A | 0.3081003 | 0.474226 | 0.000579 | 1 |
| FAM58A | 0.1844591 | 0.0437219 | 0.5958925 | 1 |
| FAM63B | 0.3555903 | 0.7449678 | 0.0148225 | 1 |
| FAM83C-AS1 | 0.078261 | 0.2132234 | 0.0326336 | 1 |
| FANCI | 0.5582084 | 0.9136571 | 9.83E-06 | 1 |
| FAXDC2 | 0.5997322 | 0.0899247 | 0.0069366 | 1 |
| FBXL15 | 0.4375507 | 0.0207965 | 0.6202673 | 1 |
| FCHO1 | 0.8452606 | 0.0046872 | 0.7259084 | 1 |
| FES | 0.2229515 | 0.3601275 | 0.0028418 | 1 |
| FH | 0.758133 | 0.8249432 | 0.0099795 | 1 |
| FKBP4 | 0.9688636 | 0.280173 | 0.015605 | 1 |
| FLJ22184 | 0.0332127 | 0.7188644 | 0.2705955 | 1 |
| FLJ38717 | 0.8999095 | 0.0514923 | 0.0358474 | 1 |
| FOCAD | 0.0126378 | 0.3378143 | 0.1947506 | 1 |
| FOXM1 | 0.6708112 | 0.709156 | 0.0003345 | 1 |
| FOXO6 | 0.8861927 | 0.3928816 | 0.0058062 | 1 |
| FSHB | 0.1345782 | 0.0465121 | 0.5934563 | 1 |
| GINS1 | 0.4986685 | 0.8861922 | 0.0064946 | 1 |
| GLB1L2 | 0.1368523 | 0.1323333 | 0.0010552 | 1 |
| GLTSCR1L | 0.5523907 | 0.1002263 | 0.0260003 | 1 |
| GLYCTK | 0.5817652 | 0.5181345 | 0.0249289 | 1 |
| GOLGB1 | 0.5817652 | 0.9757803 | 6.34E-05 | 1 |
| GOT1L1 | 0.2100552 | 0.7384139 | 0.0095835 | 1 |
| GPATCH2L | 0.0381594 | 0.4742241 | 0.7514557 | 1 |
| GPR27 | 0.8725095 | 0.1194684 | 0.0130666 | 1 |
| GPRASP1 | 0.5582084 | 0.1368523 | 0.0133968 | 1 |
| GSTP1 | 0.9861584 | 0.1486594 | 0.0149445 | 1 |
| HDGF | 0.8725095 | 0.4074549 | 0.0261425 | 1 |
| HDGFRP2 | 0.0339355 | 0.6240693 | 0.1294082 | 1 |
| HEATR2 | 0.4299031 | 0.0054342 | 0.4075481 | 1 |
| HEATR5A | 0.8317034 | 0.1066581 | 0.0103988 | 1 |
| HJURP | 0.4299071 | 0.7384128 | 0.000636 | 1 |
| HMGA1 | 0.4822996 | 0.4796001 | 0.0024574 | 1 |
| HMGB3 | 0.5877262 | 0.7059319 | 0.0015998 | 1 |
| HNRNPAB | 0.7846583 | 0.7123696 | 0.0052722 | 1 |
| ICMT | 0.6771461 | 0.033572 | 0.8036665 | 1 |
| IDH2 | 0.89991 | 0.6488269 | 0.0003916 | 1 |
| IDNK | 0.8452612 | 0.0665677 | 0.0453208 | 1 |
| IGF2BP3 | 0.5294306 | 0.501427 | 0.0149663 | 1 |
| IGFBP4 | 0.7581257 | 0.4001118 | 0.0007207 | 1 |
| IL1RN | 0.4769089 | 0.0377585 | 0.1248089 | 1 |
| ITM2B | 0.3976645 | 0.2022364 | 0.0143936 | 1 |
| ITPK1 | 0.6118479 | 0.0160809 | 0.1214417 | 1 |
| IVD | 0.0369659 | 0.7515413 | 0.688323 | 1 |
| JMJD8 | 0.1573919 | 0.0098634 | 0.116787 | 1 |
| KCNH2 | 0.6240708 | 0.8317041 | 0.008934 | 1 |
| KIAA0101 | 0.6057749 | 0.3356307 | 4.41E-05 | 1 |
| KIAA0408 | 0.0437228 | 0.1612517 | 0.3457881 | 1 |
| KIAA1468 | 0.0196284 | 0.6708085 | 0.1126503 | 1 |
| KIF14 | 0.8861927 | 0.4635797 | 0.006559 | 1 |
| KIF18B | 0.6179462 | 0.9688636 | 0.0024333 | 1 |
| KIF20A | 0.8317041 | 0.6866925 | 0.0102064 | 1 |
| KIF2C | 0.7384139 | 0.417347 | 0.0034332 | 1 |
| KIFC1 | 0.7482512 | 0.0269718 | 0.1843557 | 1 |
| KLHL3 | 0.4401131 | 0.3488555 | 0.003297 | 1 |
| KLHL42 | 0.2433365 | 0.0899204 | 0.0151288 | 1 |
| KRCC1 | 0.4426842 | 0.078261 | 0.001343 | 1 |
| KRT15 | 0.0086916 | 0.1830269 | 0.7903337 | 1 |
| LEPREL2 | 0.2246006 | 0.6708112 | 0.0039324 | 1 |
| LINC00615 | 0.529427 | 0.4850054 | 0.0193322 | 1 |
| LINC00663 | 0.0377594 | 0.2650101 | 0.0581648 | 1 |
| LINC00865 | 0.1462462 | 0.5582084 | 0.0003037 | 1 |
| LINC00899 | 0.3248622 | 0.0016157 | 0.0791433 | 1 |
| LOC100049716 | 0.0586281 | 0.0008523 | 0.6371367 | 1 |
| LOC100130992 | 0.238122 | 0.0907465 | 0.0001312 | 1 |
| LOC100506713 | 0.794689 | 0.0393864 | 0.2602222 | 1 |
| LOC100652824 | 0.7913474 | 0.9136571 | 0.0037552 | 1 |
| LOC101928539 | 0.0450955 | 0.2295999 | 0.8503632 | 1 |
| LOC101930026 | 0.0665677 | 0.0238452 | 0.2108994 | 1 |
| LOC101930415 | 0.417347 | 0.0093794 | 0.9919558 | 1 |
| LOC151174 | 0.6550784 | 0.0361883 | 0.3131132 | 1 |
| LOC283861 | 0.8317041 | 0.6930838 | 0.006834 | 1 |
| LOC284648 | 0.9550375 | 0.2919248 | 0.0156375 | 1 |
| LOC80154 | 0.16386 | 0.1368506 | 0.0006483 | 1 |
| LPPR3 | 0.2879713 | 0.0354238 | 0.2829871 | 1 |
| LPXN | 0.0155164 | 0.2100532 | 0.0756596 | 1 |
| LRRC2 | 0.0484537 | 0.2504224 | 0.326039 | 1 |
| LRRC61 | 0.2576404 | 0.0739554 | 0.0386558 | 1 |
| LYAR | 0.1236415 | 0.2007415 | 0.0174406 | 1 |
| MAP1A | 0.5967187 | 1.33E-06 | 0.2422229 | 1 |
| MAP1LC3B2 | 0.447858 | 0.4796001 | 0.0213318 | 1 |
| MAPK8IP3 | 0.0354238 | 0.2959147 | 0.1915391 | 1 |
| MCEE | 0.6488269 | 0.5466065 | 0.0184894 | 1 |
| MCM2 | 0.5237669 | 0.2229515 | 0.0227483 | 1 |
| MCM3 | 0.3510892 | 0.4742241 | 0.0102463 | 1 |
| MCM4 | 0.7059319 | 0.5582084 | 0.0182547 | 1 |
| MEGF9 | 0.821568 | 0.4504567 | 0.0349173 | 1 |
| METTL1 | 0.6333037 | 0.9930791 | 0.0020148 | 1 |
| METTL20 | 0.5758309 | 0.238124 | 0.0214942 | 1 |
| MFSD12 | 0.3185069 | 0.9033437 | 0.0256521 | 1 |
| MGAT4B | 0.6739771 | 0.3248622 | 0.0295322 | 1 |
| MIAT | 0.0984481 | 0.0212797 | 0.7319904 | 1 |
| MICALCL | 0.068519 | 0.8930469 | 0.0039943 | 1 |
| MIIP | 0.5787937 | 0.0941076 | 0.0383354 | 1 |
| MINOS1P1 | 0.0325017 | 0.0536062 | 0.7825364 | 1 |
| MLEC | 0.8999091 | 0.3291389 | 0.0148687 | 1 |
| MLF2 | 0.4349888 | 0.7123842 | 0.0008997 | 1 |
| MRPS15 | 0.0958244 | 0.0966922 | 0.010088 | 1 |
| MRPS21 | 0.0474521 | 0.3019187 | 0.2679876 | 1 |
| MSTO1 | 0.0210352 | 0.6363936 | 0.4920047 | 1 |
| MTHFD2 | 0.7059319 | 0.4324477 | 0.0038764 | 1 |
| MTMR6 | 0.2540135 | 0.8930469 | 0.0161322 | 1 |
| MVB12B | 0.3040034 | 0.5125335 | 0.0201147 | 1 |
| MYBL2 | 0.8047425 | 0.9274299 | 0.0038196 | 1 |
| MYL6B | 0.3488427 | 0.7156151 | 0.0004406 | 1 |
| NAB1 | 0.1066566 | 0.453062 | 0.0028804 | 1 |
| NAP1L1 | 0.243324 | 0.5817502 | 0.0001693 | 1 |
| NCAPD2 | 0.4148588 | 0.6457089 | 0.0153753 | 1 |
| NCAPG | 0.5758326 | 0.6866925 | 0.0097511 | 1 |
| NCS1 | 0.2296081 | 0.0304491 | 0.8431615 | 1 |
| NDRG2 | 0.6676525 | 0.0021359 | 0.5211356 | 1 |
| NDUFS6 | 0.9584922 | 0.2148185 | 0.0066462 | 1 |
| NEK2 | 0.9412251 | 0.4273793 | 0.017448 | 1 |
| NHLH2 | 0.8114631 | 0.7515424 | 0.0499648 | 1 |
| NHLRC2 | 0.2576425 | 0.0210363 | 0.6783303 | 1 |
| NIPSNAP1 | 0.1236431 | 0.4025617 | 0.0142821 | 1 |
| NIPSNAP3A | 0.8384767 | 0.0410761 | 0.8490986 | 1 |
| NLRP1 | 0.1977076 | 0.758134 | 0.0339009 | 1 |
| NLRX1 | 0.0024323 | 0.8690934 | 0.0530525 | 1 |
| NME1 | 0.5640505 | 0.2364008 | 0.0061415 | 1 |
| NMNAT1 | 0.3739495 | 0.9896188 | 0.0003107 | 1 |
| NOC4L | 0.4074549 | 0.0746618 | 0.0483795 | 1 |
| NOL6 | 0.1830269 | 0.0198576 | 0.2764314 | 1 |
| NOP10 | 0.3600662 | 0.8998973 | 0.0009381 | 1 |
| NOP14 | 0.1612517 | 0.1612517 | 0.0060105 | 1 |
| NOP2 | 0.2450952 | 0.2468606 | 0.0001387 | 1 |
| NR1H3 | 0.1414894 | 0.9896188 | 0.0021959 | 1 |
| NRXN3 | 0.7059319 | 0.2650101 | 0.0068042 | 1 |
| NUDC | 0.1312134 | 0.1225807 | 0.0030769 | 1 |
| NUDT1 | 0.3601254 | 0.3510913 | 0.000205 | 1 |
| NUF2 | 0.7846722 | 0.6803234 | 0.0121105 | 1 |
| NUSAP1 | 0.3206134 | 0.8861918 | 0.0011023 | 1 |
| ODC1 | 0.8622659 | 0.4298889 | 0.0032404 | 1 |
| OPTC | 0.3143127 | 0.0134298 | 0.4541749 | 1 |
| ORAI3 | 0.5582084 | 0.501427 | 0.0090384 | 1 |
| PAFAH1B1 | 0.7581319 | 0.4986667 | 0.0322829 | 1 |
| PALM3 | 0.9550374 | 0.5817635 | 0.0095125 | 1 |
| PAPOLA | 0.4074549 | 0.2725194 | 0.0042988 | 1 |
| PARP1 | 0.1095144 | 0.1236431 | 0.025373 | 1 |
| PCNA | 0.9619481 | 0.791342 | 0.0156094 | 1 |
| PCNP | 0.9515826 | 0.073257 | 0.006481 | 1 |
| PCYOX1 | 0.1746175 | 0.718862 | 0.0418652 | 1 |
| PHGDH | 0.7980362 | 0.1902611 | 0.0085132 | 1 |
| PIGV | 0.5582084 | 0.0484537 | 0.7702269 | 1 |
| PIH1D2 | 0.1932129 | 0.3786246 | 0.0036735 | 1 |
| PIM3 | 0.3422022 | 0.5611258 | 1.56E-05 | 1 |
| PINX1 | 0.1977076 | 0.6994971 | 0.0181473 | 1 |
| PITRM1 | 0.0760859 | 0.0437228 | 0.8423443 | 1 |
| PKLR | 0.0915758 | 0.1462409 | 0.0236718 | 1 |
| PKMYT1 | 0.7482512 | 0.4223416 | 0.0298368 | 1 |
| PLA2G4F | 0.5758326 | 0.0311208 | 0.914988 | 1 |
| PLEKHF1 | 0.3466308 | 0.1858996 | 0.0207054 | 1 |
| PLEKHM3 | 0.1057213 | 0.0760859 | 0.0368511 | 1 |
| PLEKHN1 | 0.2398561 | 0.0278854 | 0.6631654 | 1 |
| PMM2 | 0.0385656 | 0.5153282 | 0.0554194 | 1 |
| PNCK | 0.0804904 | 0.3122356 | 0.0035876 | 1 |
| POLE | 0.4796001 | 0.2613073 | 0.0058659 | 1 |
| POLQ | 0.2650101 | 0.9067801 | 0.020346 | 1 |
| POP7 | 0.5153282 | 0.9515828 | 0.0227944 | 1 |
| PPIA | 0.2919015 | 0.4452478 | 0.0248162 | 1 |
| PQLC3 | 0.3334605 | 0.6363981 | 0.020214 | 1 |
| PRDX4 | 0.7880024 | 0.7384072 | 0.0291222 | 1 |
| PSMG3 | 0.2763217 | 0.2763217 | 0.0100776 | 1 |
| PSRC1 | 0.4931796 | 0.5728754 | 0.0041326 | 1 |
| PTPN22 | 0.7846722 | 0.417347 | 0.0247422 | 1 |
| PTTG1 | 0.3040013 | 0.961949 | 0.0204232 | 1 |
| PUS1 | 0.1830308 | 0.865682 | 0.0009272 | 1 |
| PWP2 | 0.4796021 | 0.0042615 | 0.1180254 | 1 |
| PXDN | 0.1174229 | 0.0076464 | 0.943729 | 1 |
| PYCR1 | 0.1144046 | 0.0058087 | 0.5214861 | 1 |
| RAB11FIP2 | 0.2312921 | 0.0058862 | 0.7247771 | 1 |
| RAB15 | 0.6676539 | 0.9619491 | 0.0166404 | 1 |
| RACGAP1 | 0.7253589 | 0.8249425 | 0.00537 | 1 |
| RAD17 | 0.9896188 | 0.1236431 | 0.0419513 | 1 |
| RAD51-AS1 | 0.2504203 | 0.5351235 | 0.0149039 | 1 |
| RAD54L | 0.8861927 | 0.9481294 | 0.0012819 | 1 |
| RAE1 | 0.3444109 | 0.5322723 | 0.0139559 | 1 |
| RAN | 0.7780071 | 0.9861584 | 0.0003126 | 1 |
| RASL10B | 0.453062 | 0.0090294 | 0.6428857 | 1 |
| RBM5 | 0.9619491 | 0.8452612 | 0.0488761 | 1 |
| RBM7 | 0.6394944 | 0.798037 | 0.0272028 | 1 |
| RDX | 0.5466047 | 0.7123866 | 0.027882 | 1 |
| RECQL4 | 0.4504567 | 0.3378121 | 0.0110263 | 1 |
| REEP4 | 0.8452612 | 0.8047433 | 0.0348568 | 1 |
| REEP5 | 0.0672098 | 0.8317019 | 0.0261367 | 1 |
| RETSAT | 0.6771407 | 0.920539 | 0.0282628 | 1 |
| RIPK1 | 0.7156223 | 0.0552387 | 0.0020937 | 1 |
| RNASE4 | 0.4074549 | 0.1323333 | 0.0346989 | 1 |
| RNF185-AS1 | 0.2084779 | 0.1164072 | 0.0176153 | 1 |
| RNF216 | 0.0249442 | 0.2650101 | 0.0720677 | 1 |
| RNFT1 | 0.72536 | 0.2999388 | 0.0053638 | 1 |
| RNFT2 | 0.1977076 | 0.5817652 | 0.0007405 | 1 |
| RP11-173M1.8 | 0.2364049 | 0.0072606 | 0.3296899 | 1 |
| RP11-295G20.2 | 0.0949614 | 0.453062 | 0.0196698 | 1 |
| RP11-395B7.7 | 0.8114631 | 0.0149703 | 0.6507808 | 1 |
| RP11-998D10.7 | 0.0369667 | 0.1323333 | 0.6219256 | 1 |
| RPL36AL | 0.8148114 | 0.447818 | 0.0016065 | 1 |
